# Marine Inspired 2-(5-Halo-1*H*-indol-3-yl)-*N*,*N*-dimethylethanamines as Modulators of Serotonin Receptors: An Example Illustrating the Power of Bromine as Part of the Uniquely Marine Chemical Space

**DOI:** 10.3390/md15080248

**Published:** 2017-08-09

**Authors:** Mohamed A. Ibrahim, Abir T. El-Alfy, Kelly Ezel, Mohamed O. Radwan, Abbas G. Shilabin, Anna J. Kochanowska-Karamyan, Howaida I. Abd-Alla, Masami Otsuka, Mark T. Hamann

**Affiliations:** 1 Department of Pharmacognosy, The University of Mississippi, University, MS 38677, USA; mmibrahi@olemiss.edu (M.A.I.); SHILABIN@mail.etsu.edu (A.G.S.); anna.karamyan@ttuhsc.edu (A.J.K.-K.); 2 National Center for Natural Products Research, the University of Mississippi, University, MS 38677, USA; 3 Department of Chemistry of Natural Compounds, National Research Center, Dokki 12622, Cairo, Egypt; mohamedosman251@gmail.com (M.O.R.); howaida_nrc@yahoo.com (H.I.A.-A.); 4 Biopharmaceutical Sciences Department, Medical College of Wisconsin Pharmacy School, Milwaukee, WI 53226, USA; aelalfy@mcw.edu; 5 Department of Pharmacology, The University of Mississippi, University, MS 38677, USA; dionysus21@yahoo.com; 6 Department of Bioorganic Medicinal Chemistry, Faculty of Life Sciences, Kumamoto University, Chuo-ku, Kumamoto 862-0973, Japan; motsuka@gpo.kumamoto-u.ac.jp; 7 Department of Chemistry, East Tennessee State University, Johnson City, TN 37614, USA; 8 Department of Pharmaceutical Sciences, School of Pharmacy, Texas Tech University HSC, Amarillo, TX 79106, USA; 9 Department of Drug Discovery and Biomedical Sciences, Medical University of South Carolina, Charleston, SC 29425, USA

**Keywords:** serotonin receptors, psychiatric disorders, 5-Halo *N*,*N*-dimethyltryptamine

## Abstract

In previous studies, we have isolated several marine indole alkaloids and evaluated them in the forced swim test (FST) and locomotor activity test, revealing their potential as antidepressant and sedative drug leads. Amongst the reported metabolites to display such activities was 5-bromo-*N*,*N*-dimethyltryptamine. Owing to the importance of the judicious introduction of halogens into drug candidates, we synthesized two series built on a 2-(1*H*-indol-3-yl)-*N*,*N*-dimethylethanamine scaffold with different halogen substitutions. The synthesized compounds were evaluated for their in vitro and in vivo antidepressant and sedative activities using the mouse forced swim and locomotor activity tests. Receptor binding studies of these compounds to serotonin (5-HT) receptors were conducted. Amongst the prepared compounds, 2-(1*H*-indol-3-yl)-*N*,*N*-dimethyl-2-oxoacetamide (**1a**), 2-(5-bromo-1*H*-indol-3-yl)-*N*,*N*-dimethyl-2-oxoacetamide (**1d**), 2-(1*H*-indol-3-yl)-*N*,*N*-dimethylethanamine (**2a**), 2-(5-chloro-1*H*-indol-3-yl)-*N*,*N*-dimethylethanamine (**2c**), 2-(5-bromo-1*H*-indol-3-yl)-*N*,*N*-dimethylethanamine (**2d**), and 2-(5-iodo-1*H*-indol-3-yl)-*N*,*N*-dimethylethanamine (**2e**) have been shown to possess significant antidepressant-like action, while compounds **2c**, **2d**, and **2e** exhibited potent sedative activity. Compounds **2a**, **2c**, **2d**, and **2e** showed nanomolar affinities to serotonin receptors 5-HT_1A_ and 5-HT_7_. The in vitro data indicates that the antidepressant action exerted by these compounds in vivo is mediated, at least in part, via interaction with serotonin receptors. The data presented here shows the valuable role that bromine plays in providing novel chemical space and electrostatic interactions. Bromine is ubiquitous in the marine environment and a common element of marine natural products.

## 1. Introduction

Depression is the most common neuropsychiatric disorder affecting approximately 7% of Americans each year [1]. According to the National Institute of Mental Health (NIMH), every year about 40 million American adults suffer from anxiety disorders that frequently co-occur with other psychiatric illnesses, like depression [2]. Both disorders are often treated with antidepressant medications. All currently available antidepressant drugs primarily enhance the monoaminergic transmitter system by either inhibiting the catabolism or reuptake of monoamine neurotransmitters (serotonin, norepinephrine, or dopamine) thus elevating their brain levels. Unfortunately, clinically used antidepressants suffer from major drawbacks, including unfavorable adverse effects that range from weight gain to sexual dysfunction and a therapeutic lag of 4–8 weeks before a clinically relevant therapeutic effect is discernible. Anxiety disorders can also be treated with sedative medications, which have the added problem of potential abuse/addiction, and other adverse effects like drowsiness, dizziness, and headaches. Accordingly, there is a clear need for more effective and safer drugs for depression and anxiety disorders. Recently various marine natural products have been reported as valuable drug leads for neurological disorders and here we illustrate the unique activity of brominated molecules supporting the value of brominated marine natural products in the selectivity for neurological receptors and other target proteins [3,4,5].

In our recent chemical review of marine indole alkaloids as potential new drug leads for the control of depression and anxiety, we have seen the growing number of reported indole alkaloids and increased brominated functionality from various marine organisms. Many marine alkaloids are halogenated due to the presence of haloperoxidase enzymes in the marine environment. The structural similarity of endogenous amine neurotransmitters and indole alkaloids has led researchers to explore the neurological activity of such molecules [5,6,7,8,9,10,11]. Compounds like 6-bromoaplysinopsin, *N*-3′-ethylaplysinopsin, and 6-bromo-2′-de-*N*-methylaplysinopsin, isolated from *Smenospongia aurea*, were reported to display high-affinity antagonist binding for 5-HT_2C_ and 5-HT_2A_ serotonin receptors. *N*-3′-ethylaplysinopsin did not display selectivity to either of these two receptors; however, 6-bromo-2′-de-*N*-methylaplysinopsin exhibited ~40-fold selectivity to 5-HT_2C_ receptors [5].

**Figure 1:**
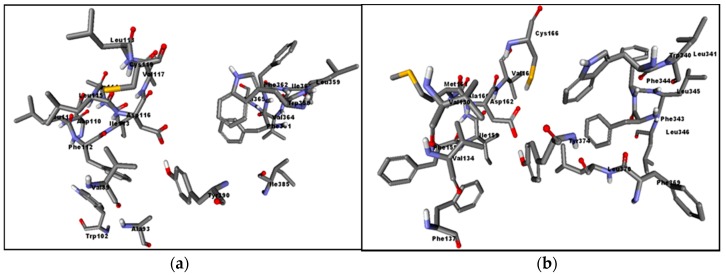
Homology-modeled active sites of serotonin receptors 5-HT_1A_ (**a**) and 5-HT_7_ (**b**) showing the dominance of hydrophobic residues (colored by element). Non-polar hydrogen atoms are omitted for clarity.

A significant number of drugs and drug candidates in clinical trials are halogenated. It is noteworthy that 50% of the top leading drugs on the market are halogenated, and halogens survive throughout the drug development process, from initial discovery to launch [12]. Halogens are included in rational drug design strategies primarily to enhance membrane permeability [13], fill spaces in the binding pocket, and decrease metabolic degradation. They can improve potency and impact target selectivity by affecting pKa and by changing conformation, lipophilicity, and hydrophobic interactions especially in hydrophobic pockets such those of serotonin receptors 5-HT_1A_ and 5-HT_7_ (Figure 1).

**Figure 2:**
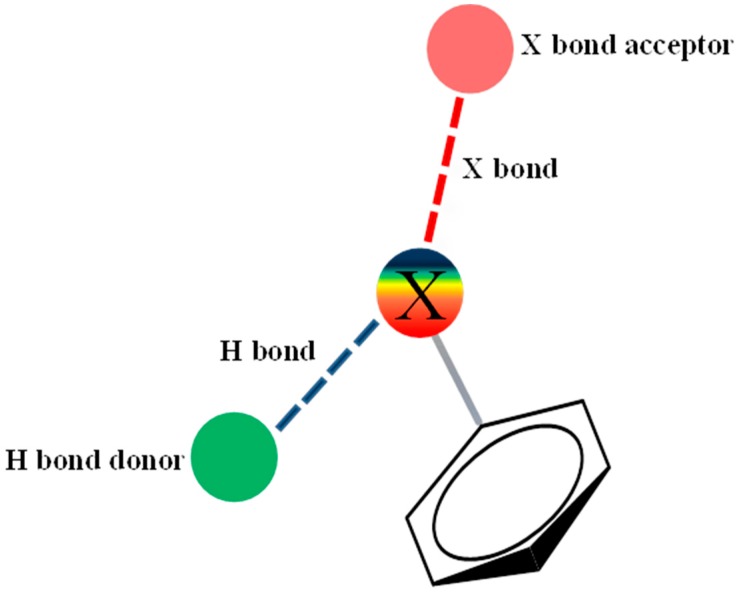
Schematic representation of halogen’s (X) dual role as an H-bond acceptor and X-bond donor in biomolecular systems. The halogen electrostatic potential is depicted gradually from negative (red) to positive (blue) demonstrating the charge anisotropic distribution (sigma hole). The X bond (red dotted line) and the H bond (blue dotted line) are directed towards an appropriate X bond acceptor and H bond donor respectively.

It is highly important to declare that halogens not only play the traditional role as H-bond acceptors, they are also endowed with establishing another intermolecular bond. This newly recognized and highly appreciated bond was recently defined as the halogen bond (X-bond) (Figure 2) [14,15] that has an emerging role in biomolecular systems. Halogen anisotropic distribution of the electron density forms a small positive electrostatic potential cap that is named the sigma hole [16,17]. Due to its exceptionally high electronegativity, fluorine is normally unable to serve as an X-bond donor, unlike other halogens. We considered fluorine incorporation into our derivatives due to its emerging application in positron emission tomography (PET) which enhances its utility for central nervous system (CNS) drug discovery. This is attributed to the favorable ^18^F half-life (109.8 min) when compared to ^13^C (20.4 min) and ^124^I (4.2 days) [18,19,20]. Taken together, we considered the incorporation of different halogen atoms in our design.

Previously, we reported the isolation of several marine indole alkaloids and evaluated them in the forced swim test (FST) and locomotor activity test, revealing their potential to become new antidepressant and sedative drug leads [3,21]. Among the compounds reported to show such activities was 5-bromo-*N*,*N*-dimethyltryptamine [3]. Because of limited supply of this natural product, a synthetic approach was adopted to prepare 5-bromo-*N*,*N*-dimethyltryptamine and its derivatives with the objective of investigating the structure activity relationships as well as conducting full dose response studies for antidepressant and sedative actions in the appropriate animal models. The current study describes the preparation of various derivatives of 2-(1*H*-indol-3-yl)-*N*,*N*-dimethylethanamine with different halogens in position five and evaluation of their activity in two animal models: forced swim and locomotor activity tests. 

The forced swim test is a well-established animal model assessing the potential clinical antidepressant action [22,23,24]. The open field locomotor activity test was used to confirm that the antidepressant action observed in the FST could not be attributed to a nonspecific stimulant activity of the tested compounds. A significant reduction in locomotor activity is usually predictive of a potential sedative action. Furthermore, the low nanomolar range affinity of some compounds towards target serotonin receptors prompted us to conduct a molecular modelling study to explore their possible binding modes and rationalize their outstanding activity.

## 2. Results and Discussion

**Figure 3:**
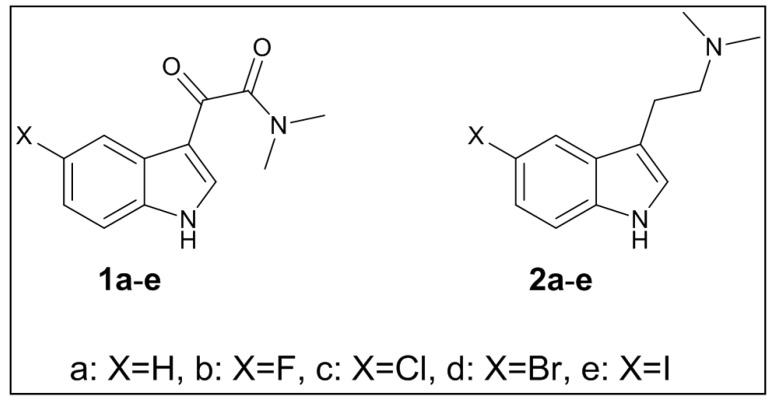
A selected series of 2-(5-halo-1H-indol-3-yl)-*N*,*N*-dimethyl-2-oxoacetamides and 2-(5-halo-1H-indol-3-yl)-*N*,*N*-dimethylethanamines.

A series of 2-(5-halo-1*H*-indol-3-yl)-*N*,*N*-dimethyl-2-oxoacetamides and 2-(5-halo-1*H*-indol-3-yl)-*N*,*N*-dimethylethanamines (Figure 3) have been synthesized via a previously reported approach with 70–90% yield [25]. We encountered problems associated with losing the halogen in the last reduction step; however, these could be eliminated by changing the solvent from tetrahydrofuran (THF), which is commonly used for these types of reactions, to 1,2-dimethoxyethane (DME) and monitoring the reaction by either gas chromatography–mass spectrometry (GC/MS) or LC/MS. The structures of the synthesized compounds have been established via 1D and 2D NMR experiments.

### 2.1. Assessment of the In Vitro Binding Affinity

Compounds **2a**, **2c**, **2d**, and **2e** were assayed for their in vitro binding to serotonin receptors in the panel of cloned human and rodent receptors, channels, and transporters available in the NIMH Psychoactive Drug Screening Program. As shown in Table 1, compounds **2a**, **2c**, **2d**, and **2e** showed high nanomolar affinity to several serotonin receptor subtypes. The highest affinity was observed towards 5-HT_1A_, 5HT_1B/1D_, 5-HT_2B_, 5-HT_6_, and 5-HT_7_ subtypes.

### 2.2. Assessment of the In Vivo Activity

Initial evaluation of analogs revealed that compounds **1a** and **1d** significantly reduced immobility in the FST (*p* < 0.001, *p* < 0.05, respectively) at the tested 20 mg/kg dose. The compounds did not significantly alter locomotor activity at this dose. Similarly, compounds **2a**, **2c**, and **2e** showed significant antidepressant-like action in the FST (*p* < 0.001, *p* < 0.01, and *p* < 0.001, respectively) when administered at the 20 mg/kg dose. Compound **2a** did not exert a significant effect on locomotor activity, while both compounds **2c** and **2e** had a significant hypolocomotive action (*p* < 0.01 and *p* < 0.001, respectively). On the other hand, compound **2b** did not exert affect immobility in the FST but significantly reduced locomotor activity (*p* < 0.01).

**Figure 4:**
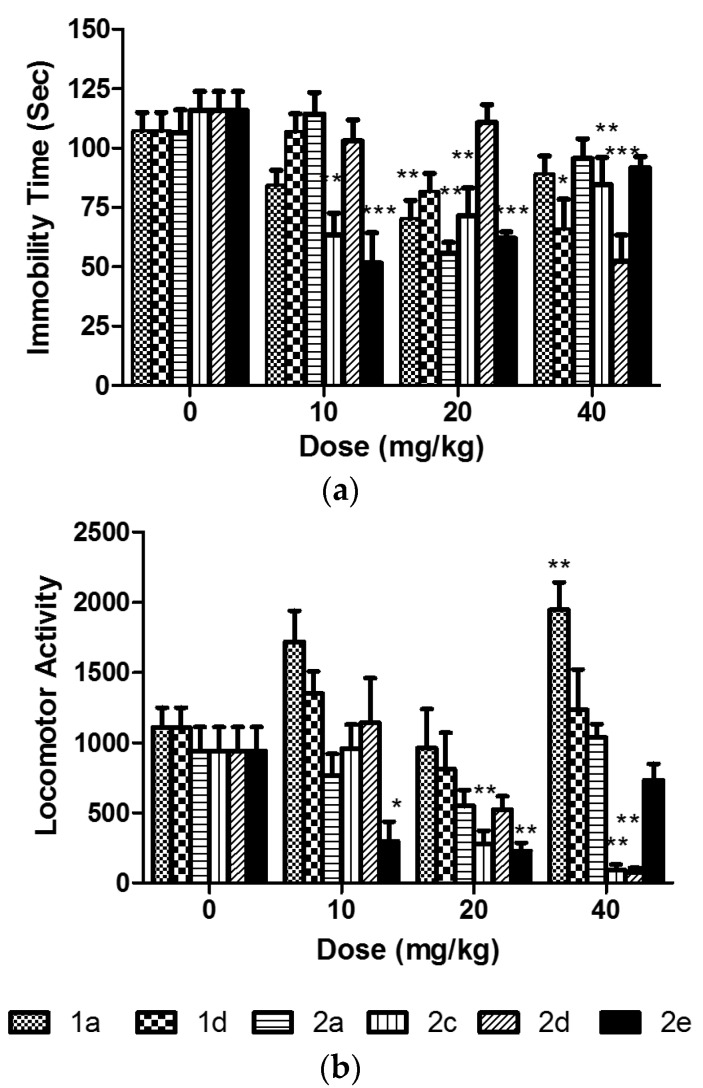
Dose response curves for the effect of compounds **1a**–**2e** in (**a**) the forced swim test and (**b**) locomotor activity. Data presented as the mean ± S.E.M. (*n* = 7–10). Data were analyzed using One Way ANOVA followed by Dunnett’s post-hoc test where * *p* < 0.05, ** *p* < 0.01, and *** *p* < 0.001 were statistically different from the vehicle control (0 mg/kg dose).

Full dose response studies for effects in FST as well as locomotor activity were conducted for compounds **1a**, **1d**, **2a**, **2c**, **2d**, and **2e** (Figure 4a,b). Results revealed that, with the exception of compound **2d**, all the tested compounds show a U-shaped dose response effect in the FST, at the tested dose range. Compound **1a** showed significant antidepressant-like action only at the 20 mg/kg dose (*p* < 0.01) and a significant increase in locomotor activity at the 40 mg/kg dose (*p* < 0.01). Compound **2c** showed a significant reduction in immobility time at the 10 (*p* < 0.01) and 20 (*p* < 0.001) mg/kg doses and significant decrease in locomotor activity at both the 20 and 40 mg/kg doses (*p* < 0.01).

Similarly, compound **2e** had significant antidepressant-like effect in the FST at 10 and 20 mg/kg (*p* < 0.001) that coincided with a significant hypolocomotive effect (*p* < 0.05 at 10 mg/kg and *p* < 0.01 at 20 mg/kg). Compound **2d**, which did not show an antidepressant-like action in the initial evaluation studies, exerted a significant reduction in immobility at 40 mg/kg (*p* < 0.001) accompanied by a significant decrease in locomotor activity at the same dose (*p* < 0.01).

As shown in Table 2, the tricyclic antidepressant desipramine, the selective serotonin reuptake inhibitor fluoxetine, and the dual dopamine and noradrenaline inhibitor bupropion, all showed dose dependent reduction in immobility times in the FST. Such effects have been previously established as a measure of antidepressant-like action. Both desipramine and fluoxetine caused significant reduction in locomotor activity indicative of their established sedative action. On the other hand, bupropion induced a significant stimulant effect. These trends are consistent with previously published literature [26,27]. 

Compound **1a** exhibited significant (*p* < 0.05) antidepressant-like activity in the FST at the 20 mg/kg dose (Figure 4a). Similarly, compound 1d caused significant (*p* < 0.05) reduction in immobility indicative of antidepressant action, but at the higher 40 mg/kg dose. Furthermore, such effect was not associated with any effect on the locomotor activity of the animals as shown in Figure 4b. Such data indicate possible antidepressant action for both compounds without potential sedative effect at the tested dose. Figure 4b shows the effect of compounds **2a**–**e** in the FST and on the locomotor activity of animals in an open field.

Compound **2a**; *N*,*N*-dimethyltryptamine (DMT) is a known hallucinogen found in psychoactive snuffs and teas used by native shamans of South America. DMT is also produced in mammalian organisms and it was recently reported to be an endogenous sigma receptor ligand [8]. In our study, compound **2a** caused significant reduction in immobility (*p* < 0.01) in the FST and a non-significant reduction in locomotor activity. The compound did not cause any locomotor stimulant action, usually associated with hallucinogenic effects, at any of the tested doses. Compounds **2c** and **2e** exhibited significant antidepressant-like action in the FST (*p* < 0.01, and *p <* 0.001, respectively) at the 20 mg/kg dose. In addition, compounds **2b**, **2c**, and **2e** caused significant reduction in locomotor activity suggesting a potential sedative effect. Compound **2b** did not show any antidepressant action in the initial evaluation, but the sedative effect was highly pronounced, thus, it was further pursued in the molecular modeling study. Such studies will help direct further research regarding the observed sedative action for this compound.

Full dose response studies for compound **1a** elicited a U-shaped dose response antidepressant-like action (Figure 4a) with the 20 mg/kg dose significantly different from the vehicle control (*p* < 0.01). A similar U-shaped response was observed in locomotor activity with a significant stimulant action evident at the 40 mg/kg dose (*p* < 0.01). All tested compounds, except compound **1d**, exhibited similar U-shaped antidepressant-like dose response curves (Figure 4a). In some cases, the lack of antidepressant action at the high dose can be partially explained by the severe sedative action observed in the locomotor activity (compound **2c**, Figure 4b). Such severe sedation can mask the antidepressant action by hindering the animal’s ability to escape or move. Further studies are needed to examine the potential sedative action of this compound. In other cases (compounds **2a** and **2e**), the trend of effect on locomotor activity does not correlate with the lack of antidepressant action (Figure 4b). The observed U-shaped dose response could possibly be attributed to activation of a separate set of pathways through the action on multiple receptors at the high dose. Thus, mechanistic studies are warranted to delineate the mechanisms underlying the observed antidepressant and sedative actions for these compounds. On the other hand, compound **1d** showed a dose-dependent response curve with significant antidepressant-like action at the 40 mg/kg dose (*p* < 0.01) and no significant effect on locomotor activity (Figure 4b).

### 2.3. Assessment of the Docking with 5-HT_7_, and 5-HT_1A_

**Figure 5:**
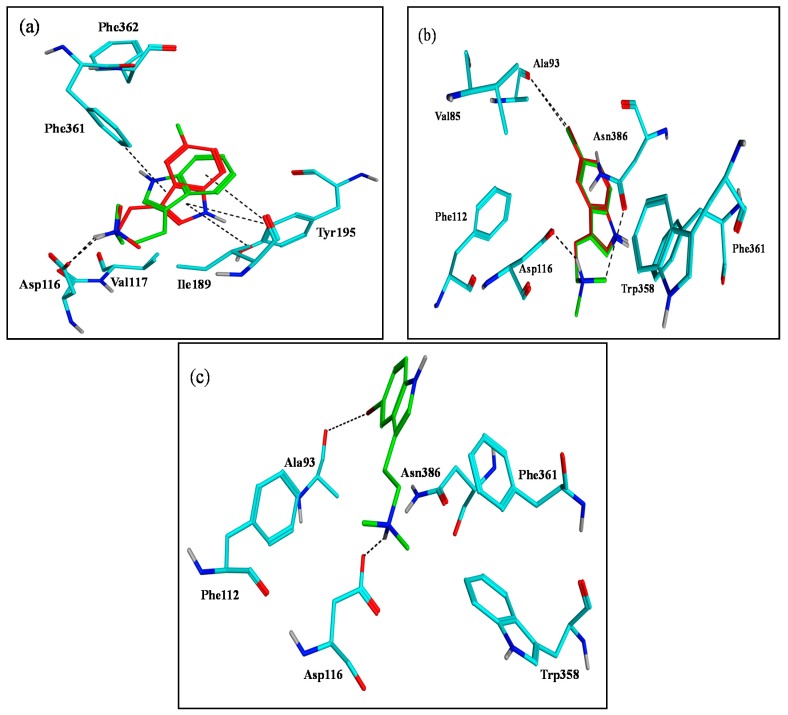
Docking interactions of compounds **2a**–**e** into 5-HT_1A_ model binding site. (**a**) Superimposed structures of **2a** (green) and **2b** (red). (**b**) Superimposed structures of **2c** (red) and **2d** (green). (**c**) Compound **2e** (green). Key binding site residues are rendered as cyan stick models.

Using the human β_2_-adrenergic G protein-coupled receptor as a template, two homology models of 5-HT_1A_ and 5-HT_7_ were generated. Throughout our molecular modelling studies, we focused primarily on compounds **2a**–**e** that have a protonated amino group as a crucial common feature for interacting with the key amino acids Asp116 and Asp162 of 5-HT_1A_ and 5-HT_7_ models, respectively [12]. Regarding 5-HT_1A_, docking result of compound **2a** revealed its ability to form a salt bridge between its protonated dimethyl amino group and Asp116 carboxylate. Its indole hydrophobic surface is buried into the hydrophobic pocket making edge to face stacking with Tyr195 and forming an arene-H interaction with Phe361 (Figure 5a). The remaining compounds with a halogen substitution have various binding modes that can explain the affinity variations. Binding of compound **2b** with 5-HT_1A_ is somewhat more favorable than **2a** in spite of having a very similar mode (Figure 5a). Fluorine is a small atom with a van der Waals radius of 1.47 Å, slightly more than the value for hydrogen at 1.20 Å, that increases the possibility of van der Waals interactions. Moreover, the fluorine inductive effect increases the polarizability of neighboring hydrogens and the N-H bond on the indole moiety [28]. On the other hand, compounds **2c** and **2d** have reoriented to adopt the same binding conformation forming an X-bond through their chloride and bromide substitutions with Ala93, as anticipated, and two H-bonds with Asp116 and Asn386 (Figure 5b). Although **2e** is bound to Ala93 via an X-bond in the same manner, unlike **2c** and **2d**, it did not form an H-bond with Asn386. Therefore, **2e** is less anchored in the pocket leading to less affinity towards 5-HT_1A_ (Figure 5c).

**Figure 6:**
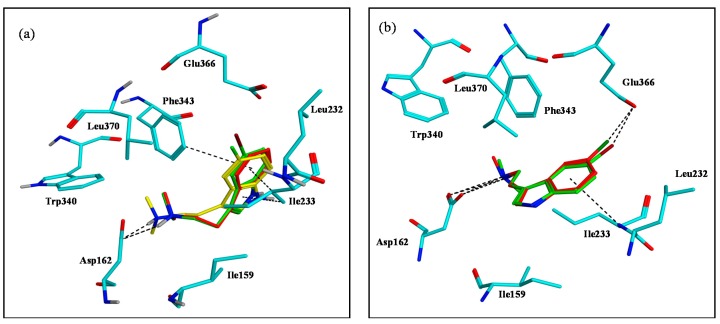
Docking interactions of compounds **2a**–**e** into 5-HT_7_ model binding site. (**a**) Superimposed structures of **2a** (green), **2b** (yellow), and **2e** (green). (**b**) Superimposed structures of **2c** (red) and **2d** (green).

Concerning docking with 5-HT_7_, the results were distinct from 5-HT_1A_. Compounds **2a**, **2b**, and **2e** had very similar binding conformations by interacting with Asp162 and embedding inside the hydrophobic pocket composed of (Leu232, Ile233, Phe343, and Leu370) (Figure 6a). Surprisingly, compound **2e** did not take the appropriate conformation to form an X-bond. On the contrary, compounds **2c** and **2d** formed X-bonds with Glu366 in addition to the Asp162 interaction. Their binding poses were further stabilized by hydrophobic interactions with the surrounding hydrophobic residues (Figure 6b). This is in good agreement with the elevation of their binding affinity in comparison with **2a**, **2b**, and **2e**. 

Furthermore, we employed a molecular operating environment (MOE) to assess toxicity or mutagenicity for all of the synthesized compounds using a rule-based method [29]. The results demonstrated the absence of any toxicophores, thus predicting a promising safety profile. Finally, we confirmed that compounds **2a**–**e** satisfy Lipinski’s rule of five as shown in Table 3.

## 3. Experimental Procedures

### 3.1. General Procedures

HPLC (High Performance Liquid Chromatography) analysis was carried out on a Waters machine equipped with a 2487 dual absorbance detector. The mass spectra were recorded using a Bruker micrOTOF (Bruker Daltonics, Bruker Inc. Billerica, MA, USA). The 1D and 2D NMR experiments were recorded on a Bruker DRX NMR spectrometer (Bruker BioSpin, Bruker Inc. Billerica, MA, USA) operating at 400 MHz and the chemical shift (δ) values were expressed in (ppm).

In vivo experiments were performed using eight week old mice. Adult male Swiss Webster mice (Harlan, IN, USA) weighing 24–31 g at the time of testing were used for the automated forced swim test. The mice were housed in groups of five with a 12 h light/12 h dark cycle. Food and water were provided *ad libitum*. All mice were randomly selected for each treatment group. 

Housing, handling, and experimental animal procedures were approved by the Institutional Animal Care and Use Committee (IACUC) of the University of Mississippi and adhered to the regulations of the National Institutes of Health Guide for Care and Use of Laboratory Animals (Protocol number 07-017).

### 3.2. Synthesis of Targeted Molecules

#### 3.2.1. The 2-(1H-Indol-3-yl)-*N*,*N*-dimethylethanamine (**2a**)

C_12_H_16_N_2_, was purified as a brownish white precipitate, ^1^H NMR (MeOD): δ = 1.61 (s, 6H), 1.99 (d, 2H), 2.35 (d, 2H), 6.41 (s, 1H), 6.56 (dt, 1H), 6.85 (d, 1H), 7.06 (d, 1H),^13^C NMR (MeOD) δ24.11 (t), 45.66 (q), 61.01 (t), 112.39 (d), 113.54 (s), 119.34 (s), 119.68 (d), 122.40 (d), 123.10 (d), 128.64 (s), 137.97 (s), (Supporting Information).

#### 3.2.2. The 2-(5-Floro-1H-indol-3-yl)-*N*,*N*-dimethylethanamine (**2b**)

C_12_H_15_N_2_F, was purified as a yellowish white precipitate, ^1^H NMR (MeOD), ^1^H NMR δ1.79 (s, 6H), 2.97 (d, 2H), 3.12 (d, 2H), 6.74 (dt, 1H), 7.08 (s, 1H), 7.15 (m, 2H). ^13^C NMR (MeOD) δ22.01 (t), 43.64 (q), 59.15 (t), 103.76 (d), 104.00 (s), 110.83 (s), 111.10 (d), 113.44 (d), 113.54 (d), 126.16 (s), 134.86 (s), (Supporting Information).

#### 3.2.3. The 2-(5-Chloro-1H-indol-3-yl)-*N*,*N*-dimethylethanamine (**2c**)

C_12_H_15_N_2_Cl, was purified as a yellowish white precipitate, ^1^H NMR (MeOD), ^1^H NMR δ2.36 (s, 6H), 2.70 (d, 2H), 2.91 (d, 2H), 5.93 (t, 1H), 6.99 (s, 1H), 7.24 (d, 1H), 7.47(s, 1H). ^13^C NMR (MeOD) δ22.48 (t), 43.67 (q), 59.67 (t), 110.83 (d), 111.60 (s), 117.69 (s), 118.21 (d), 120.96 (d), 121.83 (d), 127.15 (s), 136.79 (s), (Supporting Information).

#### 3.2.4. The 2-(5-Bromo-1H-indol-3-yl)-*N*,*N*-dimethylethanamine (**2d**)

C_12_H_15_N_2_Br, was purified as a yellowish white precipitate, ^1^H NMR (MeOD), ^1^H NMR δ2.191 (s, 6H), 2.48 (d, 2H), 2.75 (d, 2H), 6.95 (s, 1H), 7.07 (d, 1H), 7.130 (d, 1H), 7.56 (s, 1H). ^13^C NMR (MeOD) δ24.11 (t), 45.46 (q), 61.39 (t), 112.88 (d), 113.57 (s), 114.05 (s), 121.88 (d), 124.86 (d), 125.14 (d), 130.59 (s), 136.84 (s), (Supporting Information).

#### 3.2.5. The 2-(5-Iodo-1H-indol-3-yl)-*N*,*N*-dimethylethanamine (**2e**)

C_12_H_15_N_2_I, was purified as a yellowish white precipitate, ^1^H NMR (MeOD), ^1^H NMR δ2.21 (s, 6H), 2.53 (d, 2H), 2.82 (d, 2H), 6.93 (s, 1H), 6.99 (t, 1H), 7.22 (d, 1H), 7.42 (d, 1H). ^13^C NMR (MeOD) δ24.29 (t), 46.0 (q), 61.80 (t), 112.41 (d), 113.66 (s), 119.30 (s), 119.77 (d), 122.42 (d), 123.22 (d), 128.75 (s), 138.25 (s), (Supporting Information).

### 3.3. In Vitro Binding to Serotonin Receptors

Compounds **2a**, **2c**, **2d**, and **2e** were tested in the NIMH Psychoactive Drug Screening Program (University of North Carolina, Chapel Hill, NC, USA) in a panel of cloned human and rodent receptors, channels, and transporters (Table 1). For more experimental details, please refer to the Psychoactive Drug Screening Program (PDSP) web site https://pdspdb.unc.edu/pdspWeb/.

### 3.4. The Forced Swim Test (FST)

The FST represents a model of behavioral despair where the mice were subjected to an inescapable situation (in our case, the mice were placed in a cylinder of water). This model usually exhibits behavioral despair within 2 min of a 6 min session. The antidepressant effect is elicited as a reduction in the immobility time and continued escape attempts (swimming and climbing) [30]. Swiss Webster mice were injected intraperitoneally (i.p.) with the examined compound, vehicle (10% ethanol), or with control antidepressant bupropion, desipramine, or fluoxetine (10–40 mg/kg, i.p.), *n* = 7–10/group. Animals were instantly placed in individual locomotor chambers where their overall activity was recorded for 30 min. The mice were then individually placed in transparent plastic cylinders (height 23 cm, internal diameter 10 cm) filled with 8 cm of deionized water at 25 °C. Each individual mouse was videotaped for 6 min. Digital video outcome was then analyzed via SMART II Video Tracking System Software (San Diego Instruments, San Diego, CA, USA). This software determined the immobility in the 6 min session, where the last four minutes’ data were utilized to determine the effect. The immobility time was clarified to be the time spent by each mouse moving at a speed below 2 cm/s. This threshold speed was chosen based on previously published data and the validation of our automated system. This threshold generated similar immobility scores to those determined from manually scored tapes [31].

Compounds **1a**–**e** and **2a**–**e** were evaluated for their possible antidepressant activity in the forced swim test [32], where the compounds were initially tested at a dose of 20 mg/kg in comparison to the model molecules: 2-(5,6-di-bromo-1*H*-indol-3-yl)-*N*,*N*-dimethylethanamine and 2-(5-bromo-1*H*-indol-3-yl)-*N*,*N*-dimethylethanamine derivatives which previously showed significant antidepressant and sedative action at a similar dose. A number of clinically used antidepressants (the selective serotonin reuptake inhibitor fluoxetine, the tricyclic antidepressant desipramine, and the dual dopamine and norepinephrine inhibitor bupropion) were utilized as positive controls. 

### 3.5. The Locomotor Activity Test

Coupled to the FST, the effect of the test compound on locomotor activity was monitored to avoid any false positives resulting from stimulant action, as well as to evaluate any potential sedative action of the compound. Locomotor activity was measured using an automated activity monitoring system (San Diego Instruments, San Diego, CA, USA). Mice were acclimated to the testing environment for 30 min and then injected (i.p.) with vehicle (10% ethanol), control antidepressant, or the test compound. Each mouse was immediately placed in a Plexiglas enclosure and locomotor activity monitored for the next 30 min. Activity was recorded as interruptions of two sets of photo-beams. The data during the last 10 min of the testing period were analyzed. Immediately following the locomotor measurements (equivalent to the 30 min pretreatment time), the mice were subject to the FST as described above.

The effect on locomotor activity was also evaluated to eliminate a non-specific stimulating effect and to reveal any possible sedative activity. As shown in Table 2, the control antidepressants showed significant dose-dependent reduction in immobility consistent with their established antidepressant action. The effect on locomotor activity was different, whereby both desipramine and fluoxetine caused significant reduction in activity, while bupropion showed a significant locomotor stimulant effect. 

### 3.6. Data Analysis

All measured values were documented as mean ± S.E.M. with *n* = 7–10 animals/group. Data were validated using One Way ANOVA and Dunnett’s post hoc test to observe the significant difference with respect to the vehicle control at *p* < 0.05.

### 3.7. Homology Modeling

To construct homology models of 5-HT_1A_ and 5-HT_7_ receptors, the crystal structure of the seven helix bundle of human β_2_-adrenergic receptor Protein Data Bank (PDB code 2RH1) [33] was retrieved from Brookhaven Protein Data Bank and employed as the template. Amino acid sequences of the target receptors (P08908 for 5-HT_1A_ and P34969 for 5-HT_7_) were downloaded from the UniProt database (http://www.uniprot.org). The models were built by I-Tasser [34] and the valid models were selected according to their C-score [35]. QuickPrep protocol, implemented in Molecular Operating Environment (MOE) 2015.10, was used to assign the charge the state of ionizable residues, add hydrogens, and perform energy minimization.

### 3.8. Preparation of Ligand Structures and Docking

Three dimensional structures of the active hits were sketched by the Builder module of MOE. They were protonated, minimized, and then docked into the generated homology models using the standard docking protocol implemented in MOE as described before [36]. Ligand conformations were placed in the site with the Triangle Matcher method and ranked with the London dG scoring function. 

## 4. Conclusions

Data collected show that compounds **2a**, **2c**, **2d**, and **2e** possessed high nanomolar affinity to several serotonin receptor subtypes, particularly 5-HT_1A_, 5-HT_1B/1D_, 5-HT_2B_, 5-HT_6_, and 5-HT_7_ subtypes. It has been shown that introduction of fluorine at position 6 of *N*,*N*-dimethyltryptamine causes a 5-fold decrease in affinity toward the 5-HT_1A_ receptor [5].

Similarly, introducing fluorine at the 6 position of 5-methoxy-*N*,*N*-dimethyltryptamine decreases the 5-HT_1A_ receptor binding affinity. However fluorination of 5-methoxy-*N*,*N*-dimethyltryptamine at position 4 increases the affinity toward the 5-HT_1A_ receptor. Based on our data (Table 1), the unsubstituted *N*,*N*-dimethyltryptamine (**2a**) shows good affinity towards 5-HT_1D_ and 5-HT_1B_ similar to 5-chloro-, bromo-, and 5-iodo-*N*,*N*-dimethyltryptamine (**2c**–**e**). In addition, 5-chloro*-N*,*N*-dimethyltryptamine shows strong affinity towards 5-HT_1A_, 5-HT_2B_, and 5-HT_7_. While 5-bromo*-N*,*N*-dimethyltryptamine (**2d**) shows strong affinity towards 5-HT_1A_, 5-HT_2B_, 5-HT_6_, and 5-HT_7_. Previous research has suggested the involvement of these serotonin receptor subtypes in depression, anxiety, and migraines [37].

The utilization of 5-HT_1A_ knockout animals has resulted in enhanced anxiety in several experimental paradigms, as well as demonstrated significant increased baseline immobility in behavioral despair tests [38,39]. Moreover, selective 5-HT_1A_ agonists have shown antidepressant actions in preclinical as well as clinical testing [40,41,42,43]. The involvement of 5-HT_1B_ receptors in mood disorders has also been observed in knockout models. Such mice exhibited a decreased anxiety in open field, elevated plus maze, and tail suspension tests. On the other hand, the same mice displayed increased aggressive behavior [44,45]. 

Additionally, 5-HT_1B_ and 5-HT_1D_ receptors are well known targets for anti-migraine medications. A number of studies have examined the role of 5-HT_2B_ receptors in mood regulation. These studies showed that direct injection of the selective 5-HT_2B_ agonist, BW 723C86, into the medial amygdale results in anxiolytic effect in the rat social interaction test [46].

The attribution of 5-HT_6_ and 5-HT_7_ receptors in neuropsychiatric disorders has recently drawn attention due to the pharmacological studies that demonstrated high affinity of several antipsychotic and antidepressant agents to these two receptor subtypes [47,48]. Thus, the in vitro data suggest that the antidepressant action exerted by these compounds in vivo might be mediated via interaction with serotonin receptors. Further mechanistic studies are hence required to delineate the nature of such interactions and further establish the mechanism underlying the observed behavioral effects of these compounds.

## Figures and Tables

**Table 1:** Binding affinities of the selected compounds towards serotonin receptors.

Receptor	Compound 2a Ki (nM)	Compound 2c Ki (nM)	Compound 2d Ki (nM)	Compound 2e Ki (nM)	Controls	
					Ergotamine Ki (nM)	Methysergide Ki (nM)
5-HT_1A_	110.0 ± 17.0	5.5 ± 0.4	9.6 ± 1.1	130.0 ± 16.0	0.17	14.0
5-HT_1B_	66.0 ± 9.0	66.0 ± 5.0	19.0 ± 2.0	43.0 ± 5.0	0.3	2.5
5-HT_1D_	29.3 ± 3.7	14.0 ± 1.0	2.6 ± 0.32	8.5 ± 1.38	0.3	69.0
5-HT_1E_	>10,000	356.0 ± 34.0	398.0 ± 30.0	310.0 ± 33.0	19.0	237.0
5-HT_2B_	145.0 ± 13.0	7.8 ± 0.7	27.0 ± 1.0	98.0 ± 4.0	1.9	0.1
5-HT_3_	5,187 ± 883	1,325 ± 125	1,374 ± 212	4,486 ± 804	>10,000	>10,000
5-HT_5A_	>10,000	408.0 ± 54.0	1,038 ± 110	1,254 ± 197	-	>10,000
5-HT_6_	189.5 ± 32.5	30.0 ± 2.0.0	22.0 ± 2.0	198.0 ± 20.0	12.0	52.0
5-HT_7_	77.0 ± 16.0	7.2 ± 0.6	8.3 ± 0.9	116.0 ± 13.0	1,291	30.0

**Table 2:** Effect of control antidepressants and synthesized compounds on immobility time in mouse forced swim test and total locomotor activity.

Treatment	Immobility (sec)	Locomotor
Vehicle	121 ± 7.3	1618 ± 142
Bupropion 10 mg/kg	101 ± 10.5	2746 ± 298 *
Bupropion 20 mg/kg	80 ± 7.1 **	3564 ± 503 ***
Bupropion 40 mg/kg	58 ± 8.2 ***	5290 ± 544 ***
Fluoxetine 10 mg/kg	91 ± 10	1898 ± 132
Fluoxetine 20 mg/kg	90 ± 6.8	1293 ± 243
Fluoxetine 40 mg/kg	75.8 ± 12.9 **	143 ± 34 ***
Desipramine 10 mg/kg	112 ± 6.6	763 ± 112 **
Desipramine 20 mg/kg	81 ± 4.9 **	776 ± 265 **
Desipramine 40 mg/kg	70 ± 8.9 ***	117 ± 43 ***
Compound **1a** 10 mg/kg	84.3 ± 6.3	1717 ± 221
Compound **1a** 20 mg/kg	70.4 ± 7.8 **	958.6 ± 279
Compound **1a** 40 mg/kg	89.1 ± 7.6	1945 ± 195 **
Compound **1d** 10 mg/kg	106.9 ± 7.5	1348 ± 159
Compound **1d** 20 mg/kg	81.8 ± 7.5	812 ± 258
Compound **1d** 40 mg/kg	66 ± 12.4 *	1233 ± 286
Compound **2a** 10 mg/kg	114.3 ± 9.2	764 ± 155
Compound **2a** 20 mg/kg	55.7 ± 4.6 **	549 ± 111
Compound **2a** 40 mg/kg	95.7 ± 8.2	1036 ± 93
Compound **2c** 10 mg/kg	63.4 ± 9.3 **	956 ± 171
Compound **2c** 20 mg/kg	71.6 ± 11.7 **	275 ± 96 **
Compound **2c** 40 mg/kg	84.6 ± 11.5	92 ± 38 **
Compound **2d** 10 mg/kg	103 ± 8.9	1141 ± 317
Compound **2d** 20 mg/kg	110.8 ± 7.5	521 ± 95
Compound **2d** 40 mg/kg	52.4 ± 11.1 ***	73 ± 34 **
Compound **2e** 10 mg/kg	51.6 ± 12.8 ***	296 ± 139*
Compound **2e** 20 mg/kg	62.1 ± 2.6 ***	227 ± 59 **
Compound **2e** 40 mg/kg	91.6 ± 4.9	729 ± 119

* *p* < 0.05, ** *p* < 0.01, *** *p* < 0.001 (Dunnett’s post-hoc test versus vehicle).

**Table 3:** Lipinski properties of compounds **2a**–**e**.

Molecule	Molecular Weight	Log p	H-Donor	H-Acceptor	Rotatable Bonds
2a	188	1.8	1	1	3
2b	206	1.95	1	1	3
2c	222	2.35	1	1	3
2d	266	2.63	1	1	3
2e	314	3.15	1	1	3

## Supplementary Materials

Supplementary material to this article can be found online at www.mdpi.com/1660-3397/15/8/248/s1.
