# Supplementary material for: Marine Inspired 2-(5-Halo-1H-indol-3-yl)-N,N-dimethylethanamines as Modulators of Serotonin Receptors: An Example Illustrating the Power of Bromine as Part of the Uniquely Marine Chemical Space
Source: Mar Drugs. 2017 Aug 9;15(8):248. doi: 10.3390/md15080248 (PMC5577603; doi:10.3390/md15080248)
Supplement: Supplementary file 1 [file marinedrugs-15-00248-s001.pdf]

Marine Inspired 2-(5-Halo-1*H*-indol-3-yl)-*N*,*N*-Dimethylethanamines As Modulators of Serotonin Receptors: An Example Illustrating the Power of Bromine as part of the Uniquely Marine Chemical Space.

Mohamed A. Ibrahim [1,2,3], Abir T. El-Alfy [4,5], Kelly Ezel [5], Mohamed O. Radwan [3,6], Abbas G. Shilabin [1,7], Anna J. Kochanowska-Karamyan [1,8], Howaida I. Abd-Alla [3], Masami Otsuka [6] and Mark T. Hamann [1,2,5,9,*]

[1]Department of Pharmacognosy, the University of Mississippi, University, MS 38677, USA; mmibrahi@olemiss.edu (M.A.I.); SHILABIN@mail.etsu.edu (A.G.S.); anna.karamyan@ttuhsc.edu (A.J.K-K.); hamannm@musc.edu (M.T.H.)
[2]National Center for Natural Products Research, the University of Mississippi, University, MS 38677, USA
[3]Department of Chemistry of Natural Compounds, National Research Center, Dokki 12622, Cairo, Egypt; mohamedosman251@gmail.com (M.O.R.); howaida_nrc@yahoo.com (H.I.A-A.)
[4]Biopharmaceutical Sciences Department, Medical College of Wisconsin Pharmacy School, Milwaukee, WI 53226, USA; aelalfy@mcw.edu
[5]Department of Pharmacology, The University of Mississippi, University, MS, 38677, USA; dionysus21@yahoo.com
[6] Department of Bioorganic Medicinal Chemistry, Faculty of Life Sciences, Kumamoto University, Chuo-ku, Kumamoto 862-0973, Japan; motsuka@gpo.kumamoto-u.ac.jp
[7]Department of Chemistry, East Tennessee State University, Johnson City, TN 37614, USA
[8] Department of Pharmaceutical Sciences, School of Pharmacy, Texas Tech University HSC, Amarillo, TX 79106, USA
[9]Department of Drug Discovery and Biomedical Sciences, Medical University of South Carolina, Charleston, SC 29425, USA

***Correspondence: hamannm@musc.edu; Tel.: +1 843-876-2316; Fax: +1 843-876-2353
3-876-2353

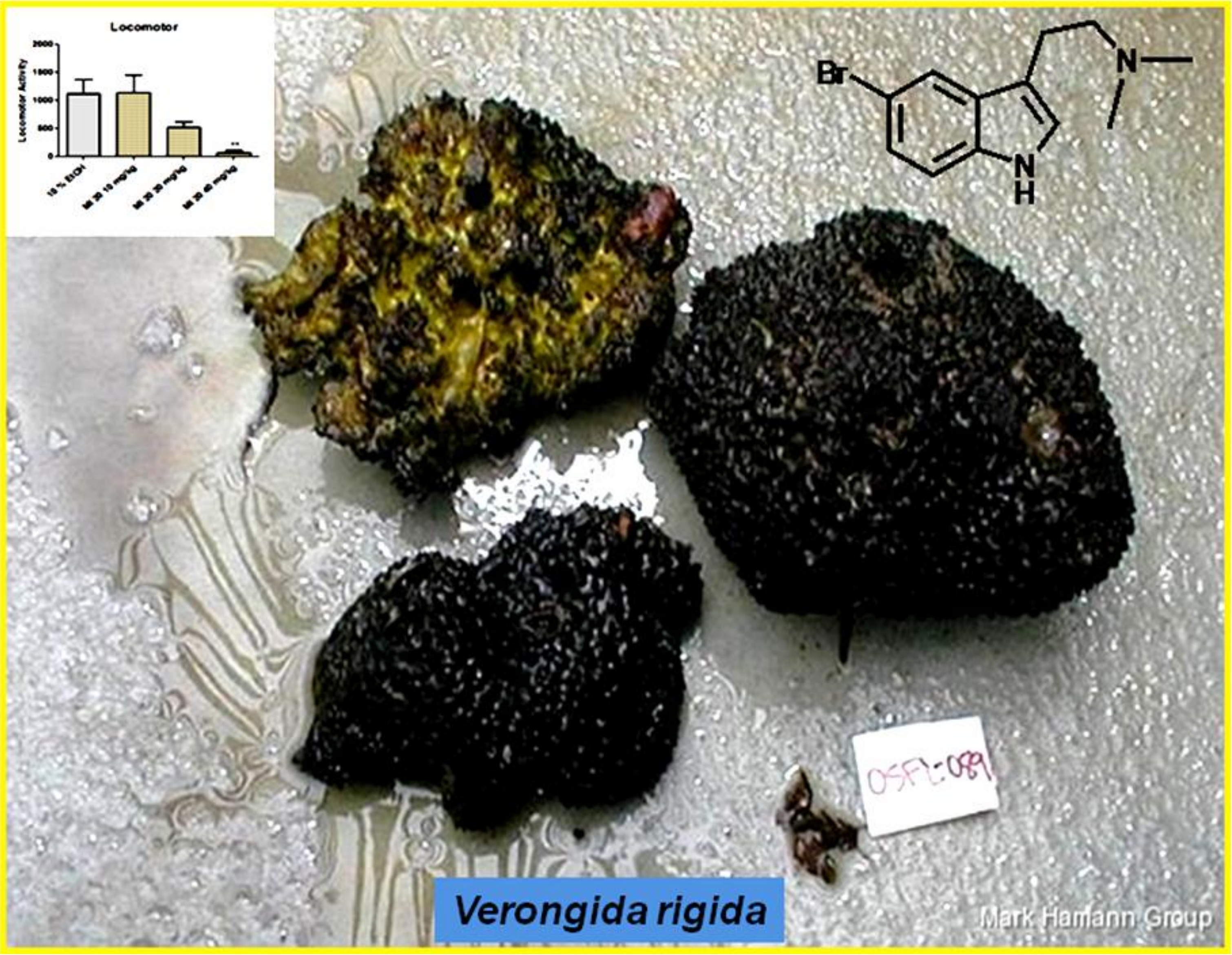

Figure 1. Picture of the sponge *Verongida rigida*

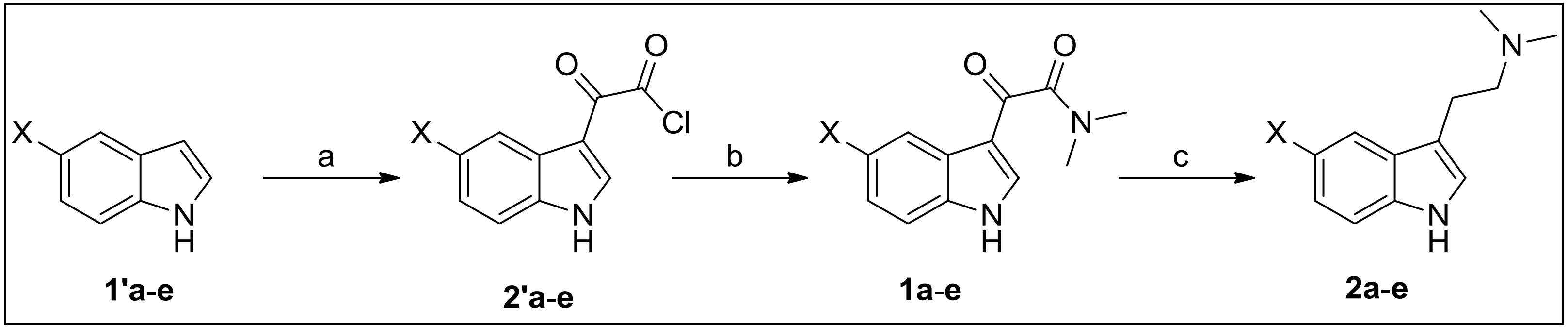

Figure 2. The synthesis of 5-haloindoledimethylethanamines. Reagents and reaction conditions: (a) Oxalyl chloride, diethyl ether, 0 ºC, 30-60 min, (80-90%); (b) Dimethylamine, 0 ºC, 30 min, then rt, 2 h, (80-90%); (c) $LiAlH_4$, DME, 0 ºC, 1 h, then rt, 2 h, then 80 ºC, 2 h, (65-75%)

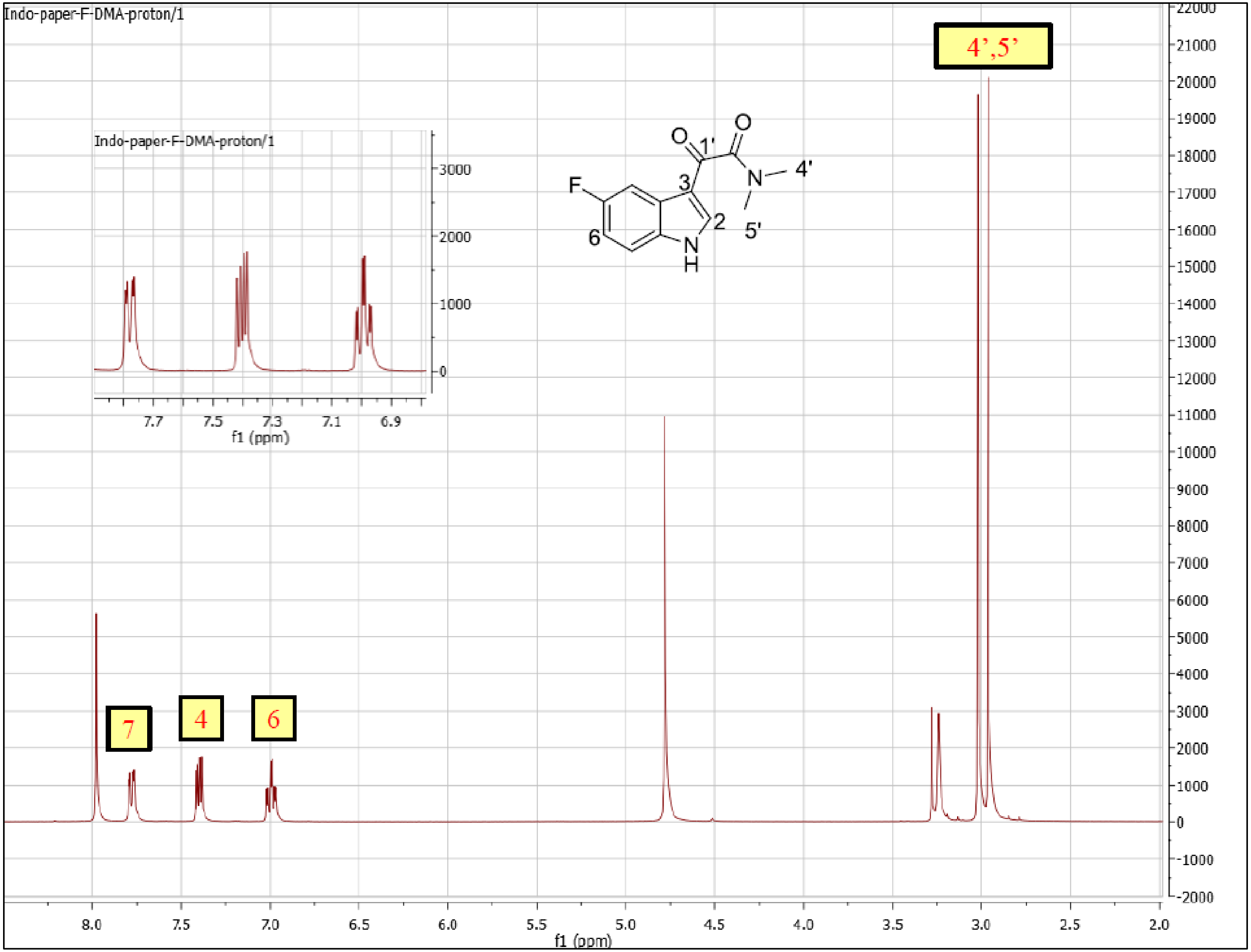

Figure 3. ^{1}H NMR spectrum of 2-(5-fluoro-1*H*-indol-3-yl)-*N*,*N*-dimethyl-2-oxoacetamide (**1b**) in methanol-d_4 (400 MHz)

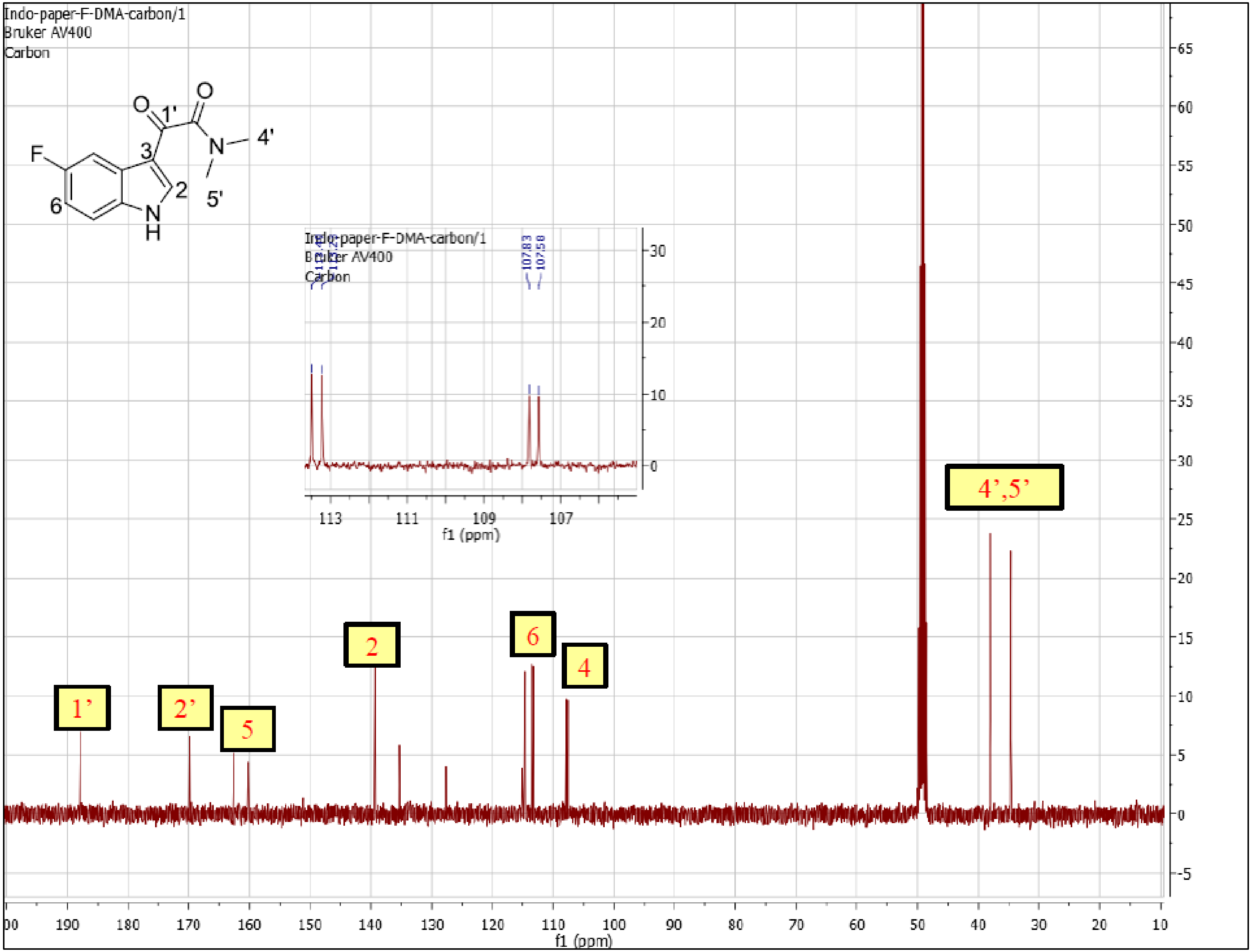

Figure 4. ^{13}C NMR spectrum of 2-(5-fluoro-1*H*-indol-3-yl)-*N*,*N*-dimethyl-2-oxoacetamide (**1b**) in methanol-d_4 (400 MHz)0

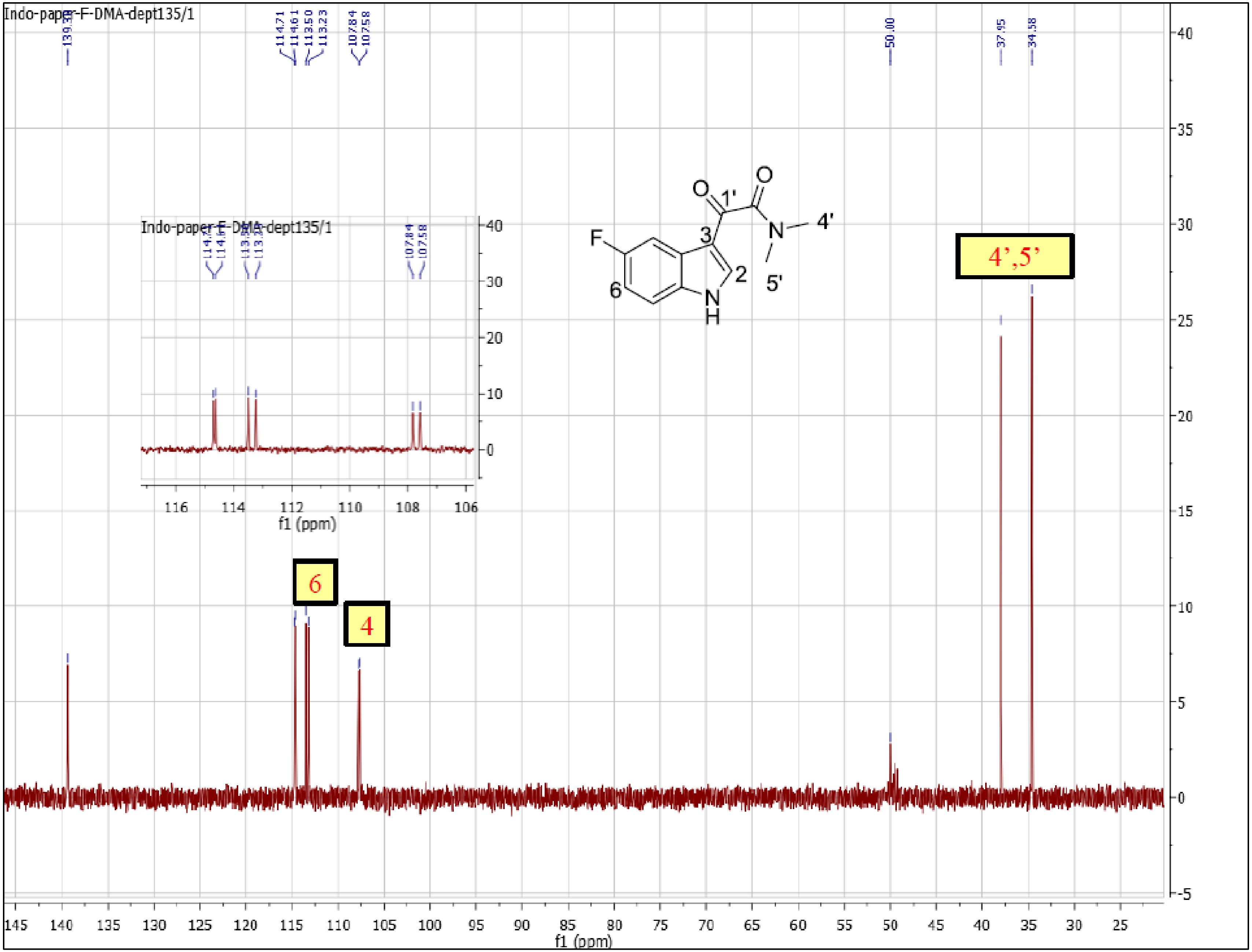

Figure 5. 135º DEPT spectrum of 2-(5-fluoro-1*H*-indol-3-yl)-*N*,*N*-dimethyl-2-oxoacetamide (**1b**) in methanol-d_4 (400 MHz)

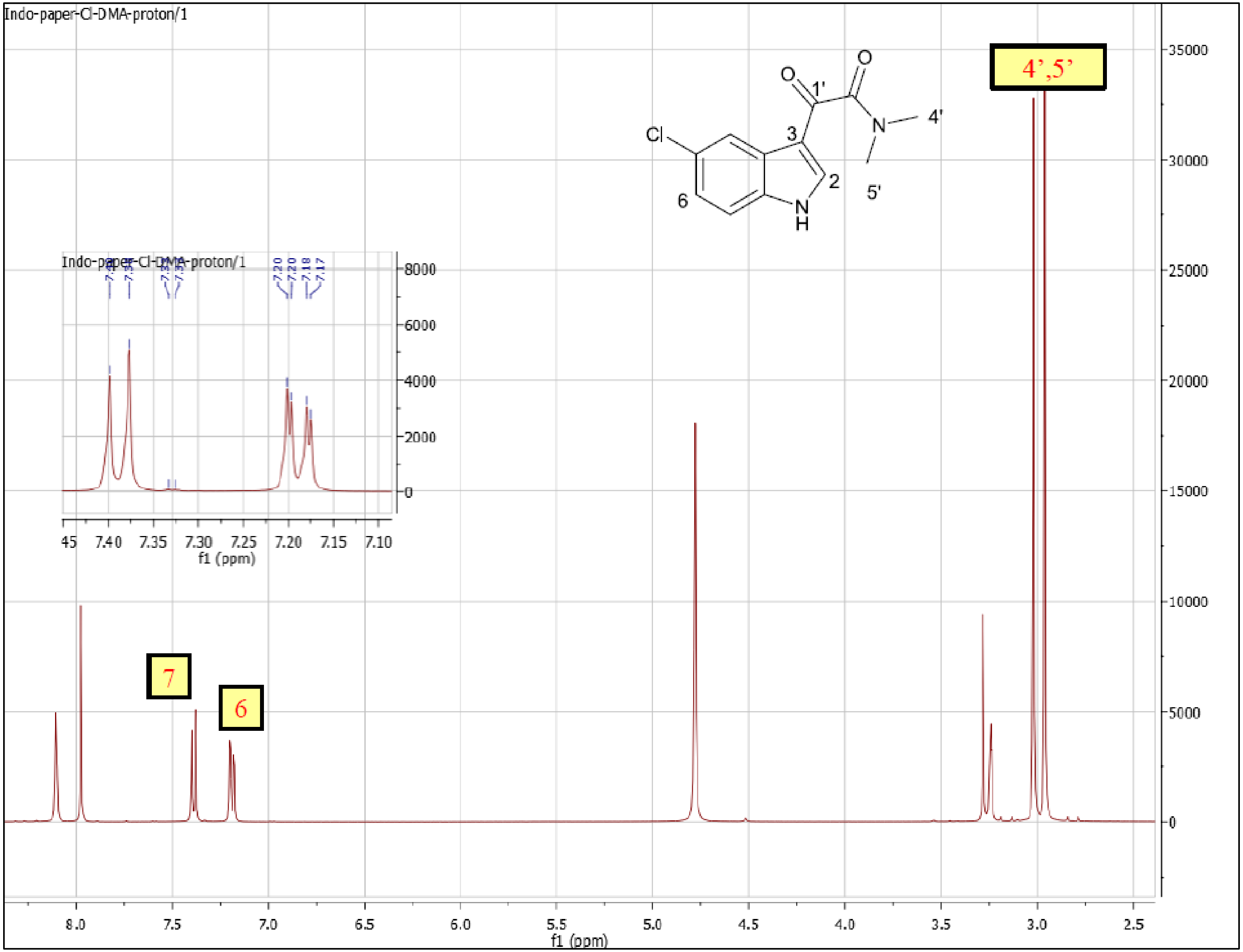

Figure 6. ^{1}H NMR spectrum of 2-(5-chloro-1*H*-indol-3-yl)-*N*,*N*-dimethyl-2-oxoacetamide (**1c**) in methanol-d_4 (400 MHz)

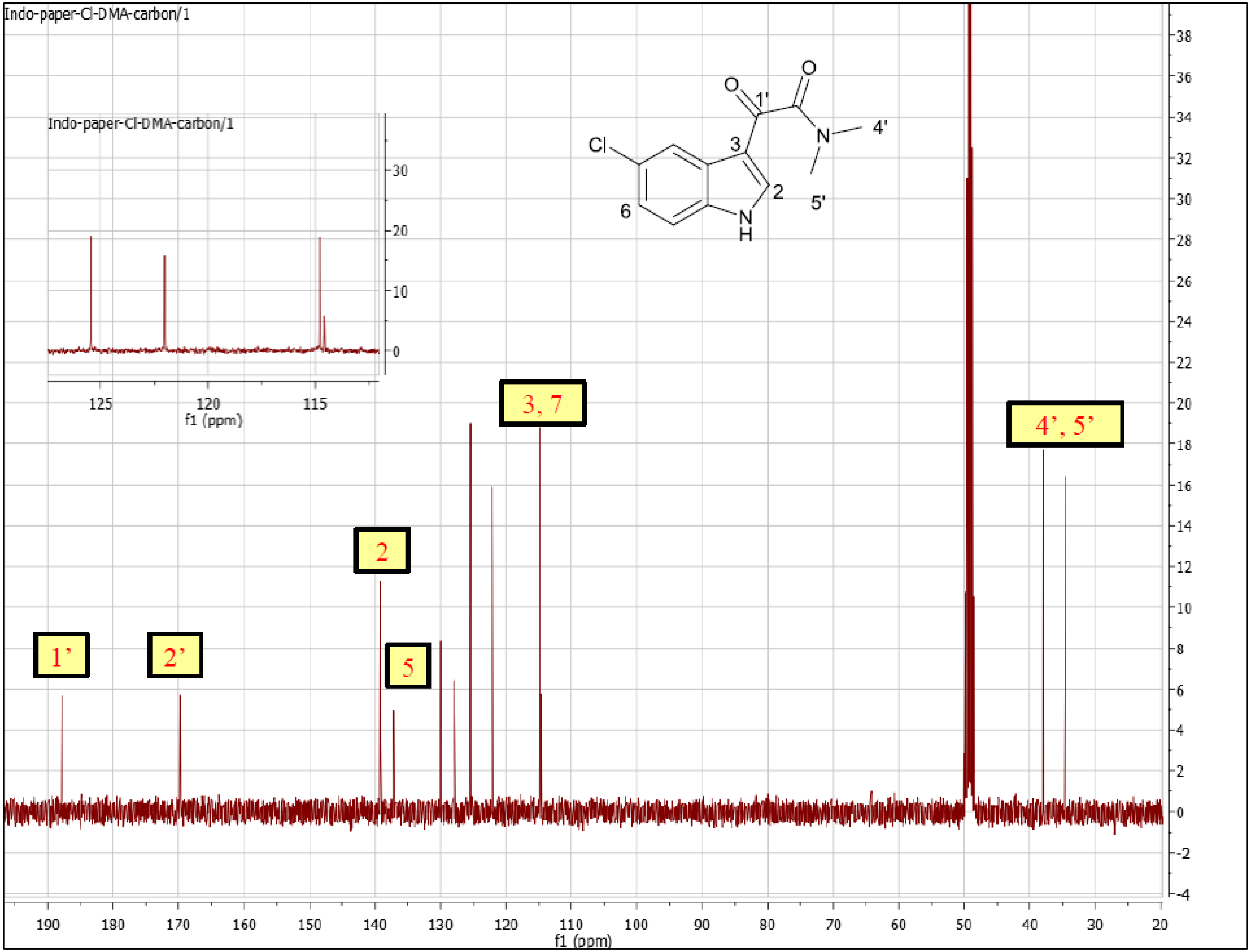

Figure 7. ^{13}C NMR spectrum of 2-(5-chloro-1*H*-indol-3-yl)-*N*,*N*-dimethyl-2-oxoacetamide (**1c**) in methanol-d_4 (400 MHz)

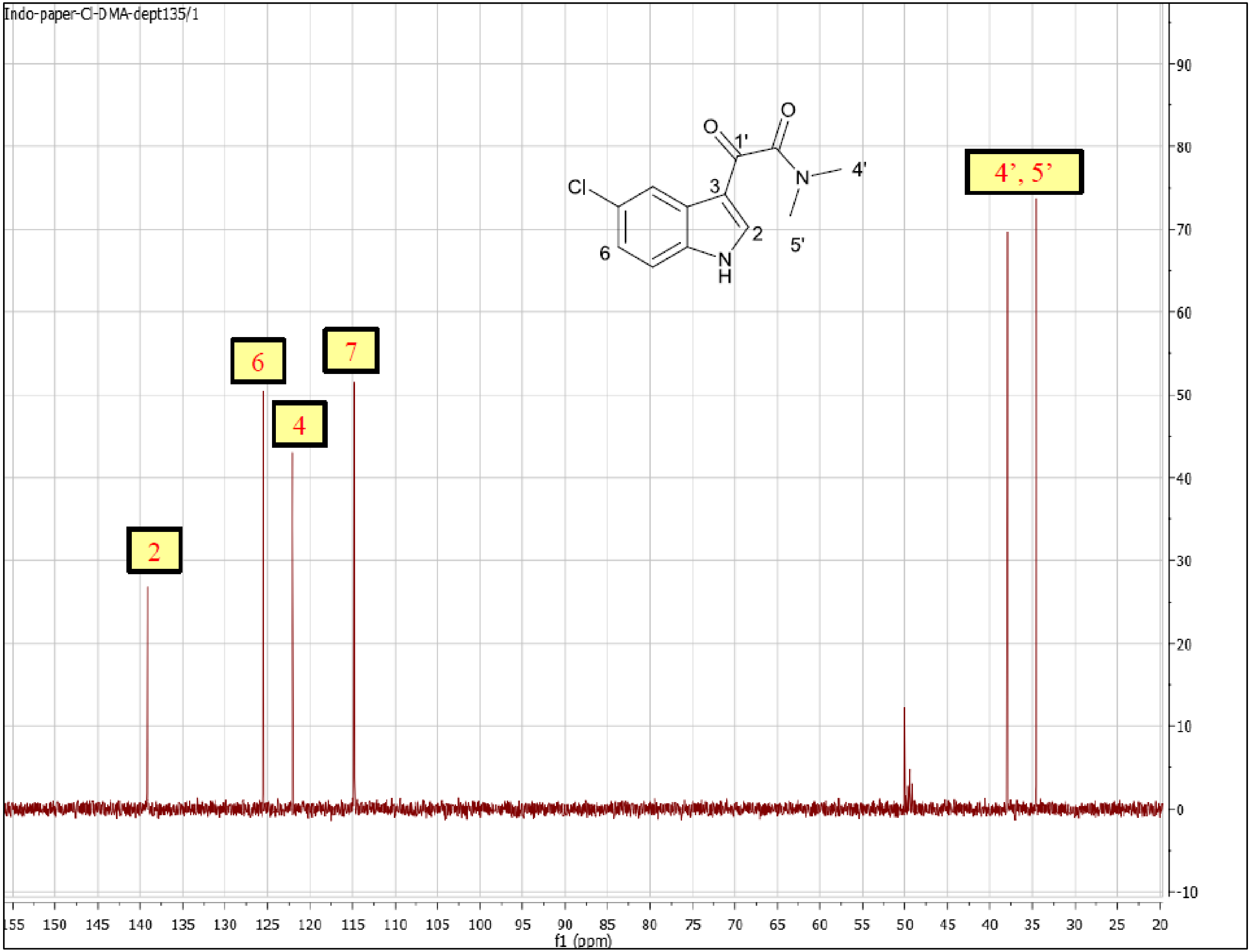

Figure 8. 135º DEPT spectrum of 2-(5-chloro-1*H*-indol-3-yl)-*N*,*N*-dimethyl-2-oxoacetamide (**1c**) in methanol-d_4 (400 MHz)

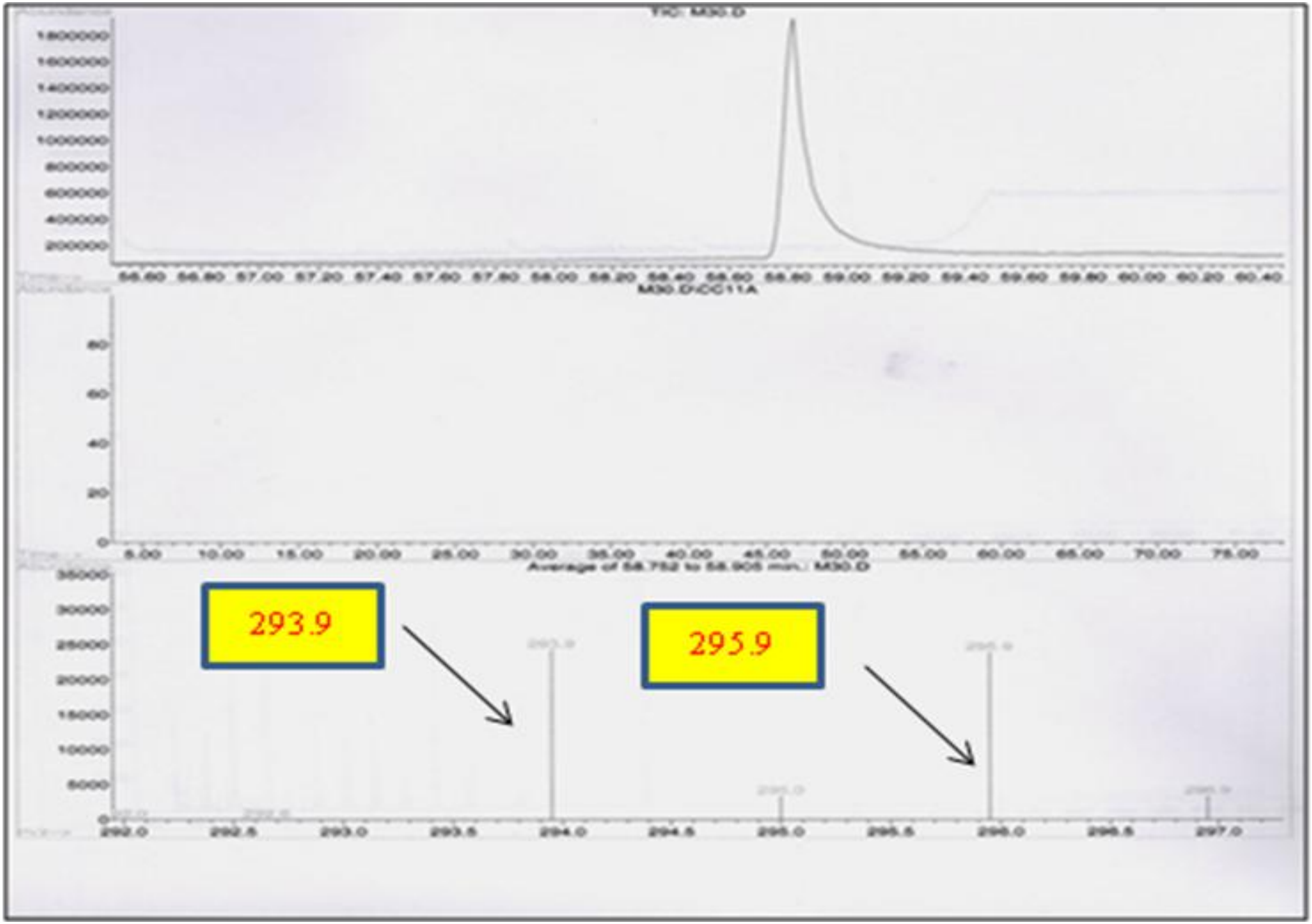

Figure 9. GC/MS chromatogram of 2-(5-bromo-1*H*-indol-3-yl)-*N*,*N*-dimethyl-2-oxoacetamide (**1d**)

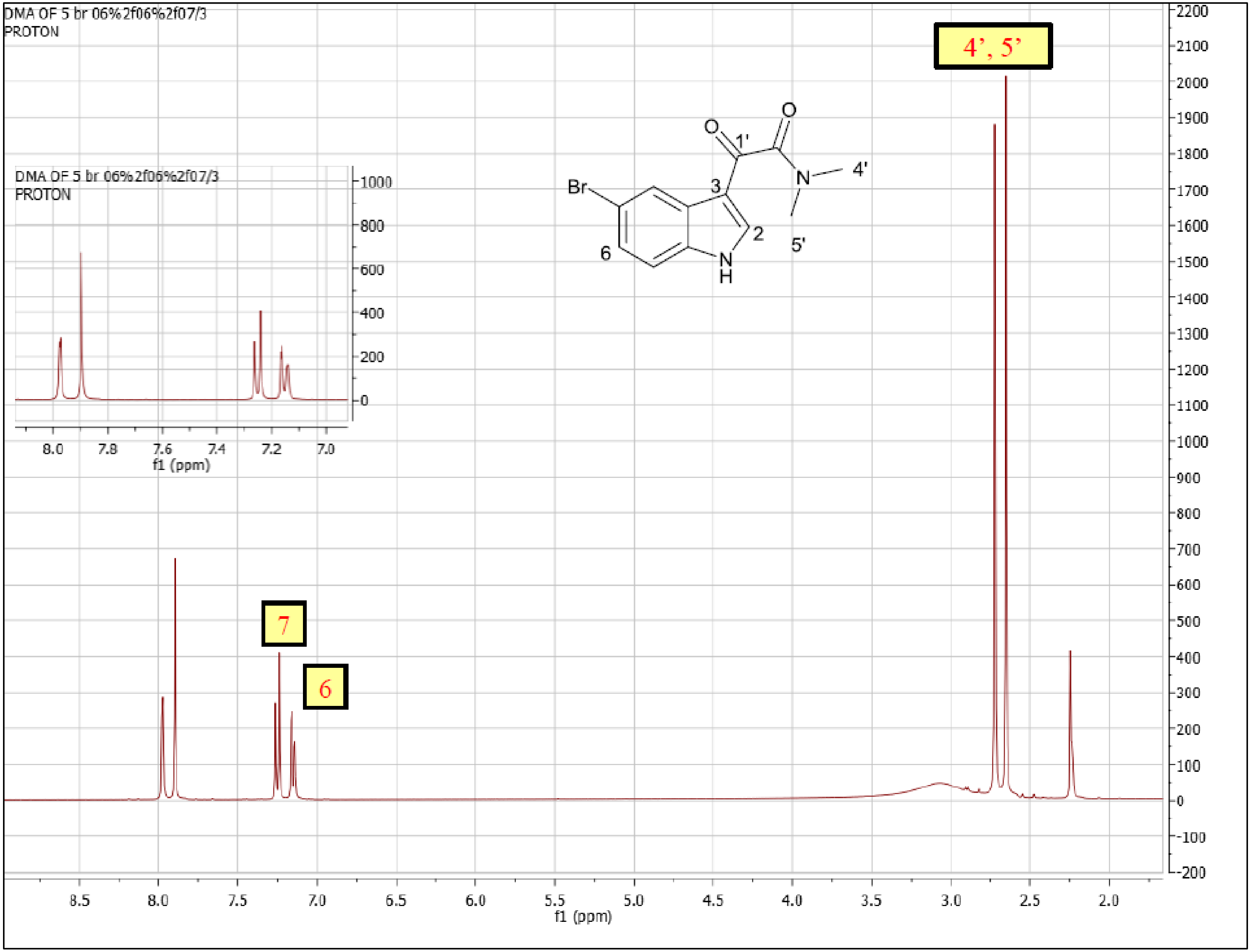

Figure 10. ^{1}H NMR spectrum of 2-(5-bromo-1*H*-indol-3-yl)-*N*,*N*-dimethyl-2-oxoacetamide (**1d**) in $CDCl_3$ (400 MHz)

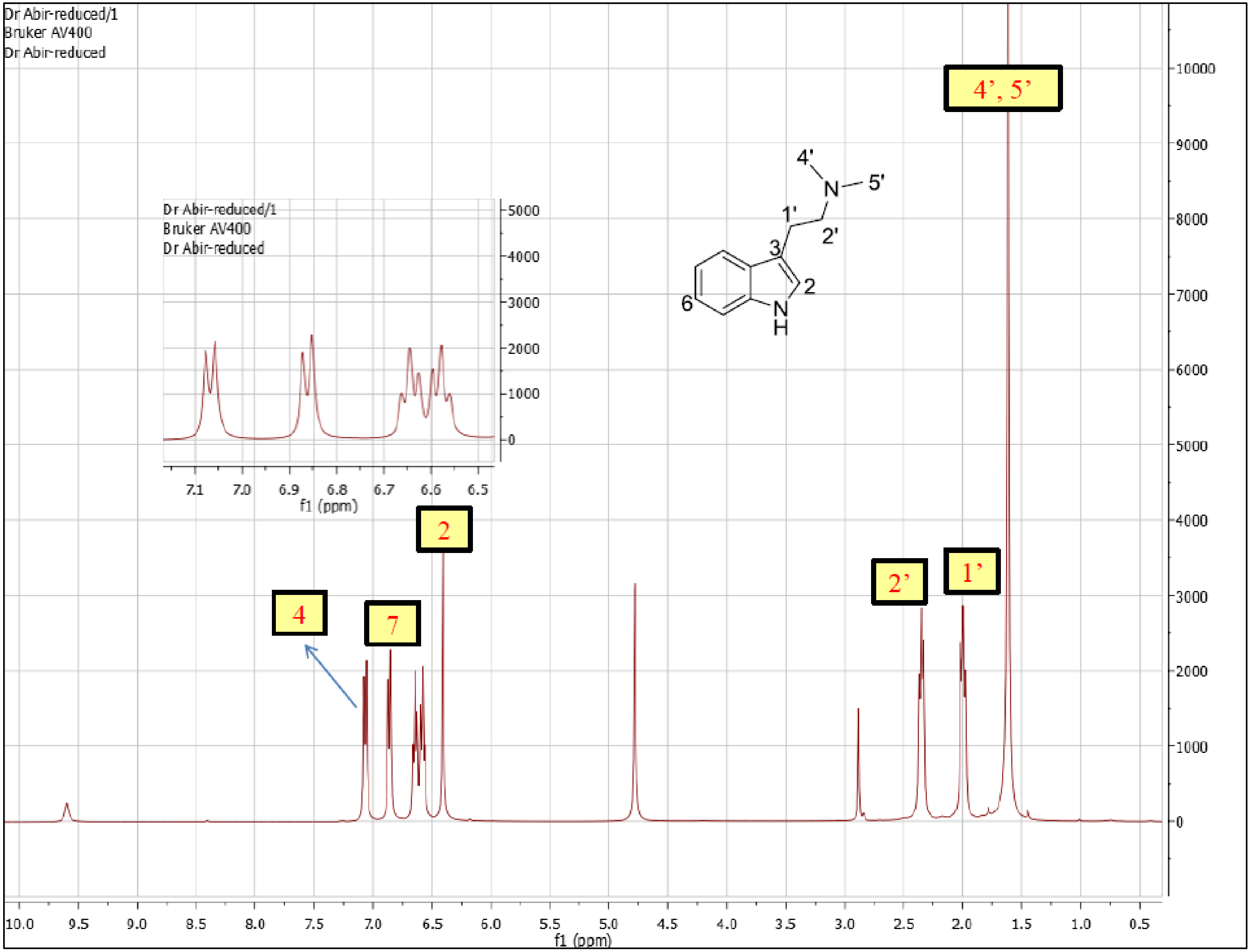

Figure 11. ^{1}H NMR spectrum of 2-(1*H*-indol-3-yl)-*N*,*N*-dimethylethanamine (**2a**) in methanol-d_4 (400 MHz)

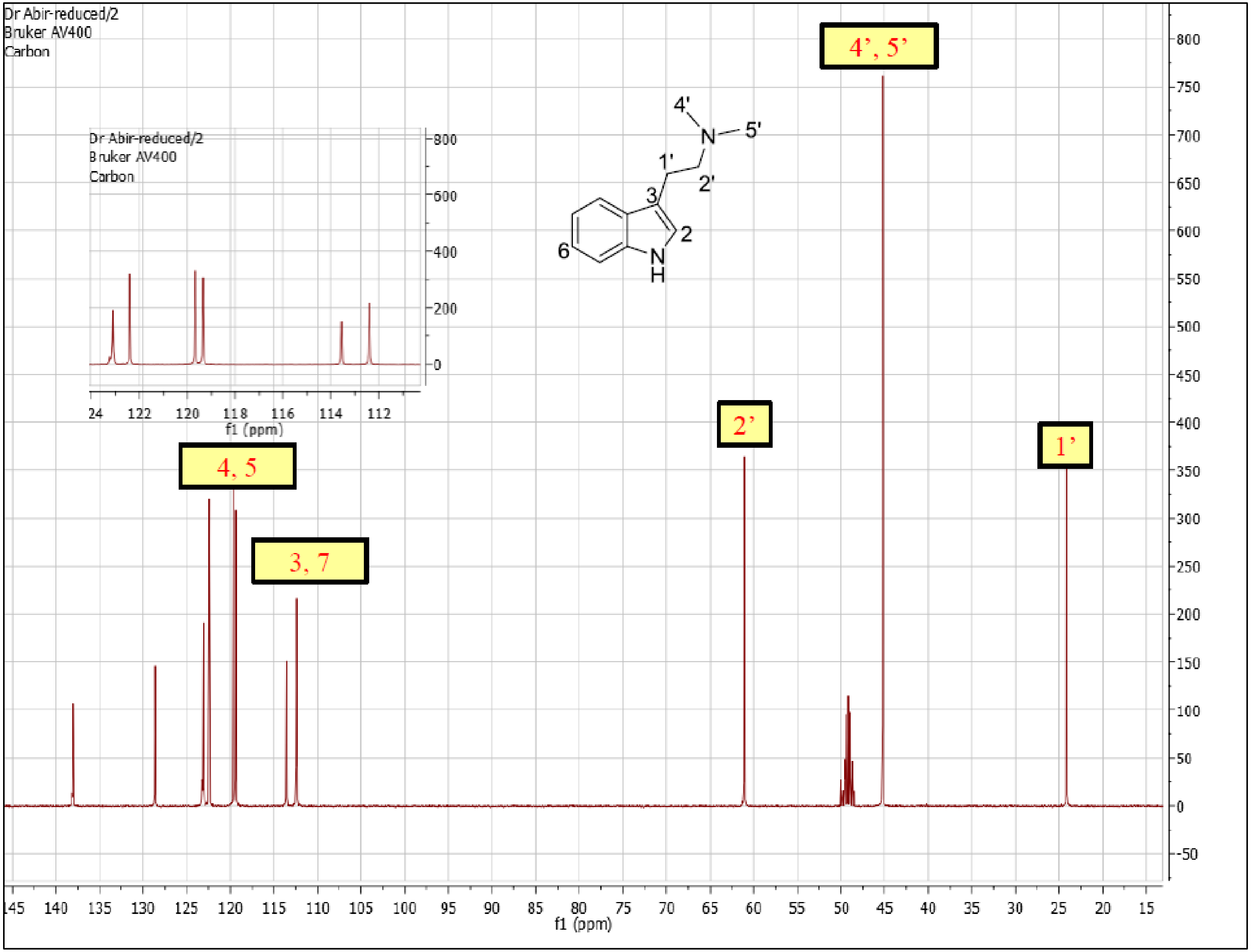

Figure 12. ^{13}C NMR spectrum of 2-(1*H*-indol-3-yl)-*N*,*N*-dimethylethanamine (**2a**) in methanol-d_4 (400 MHz)

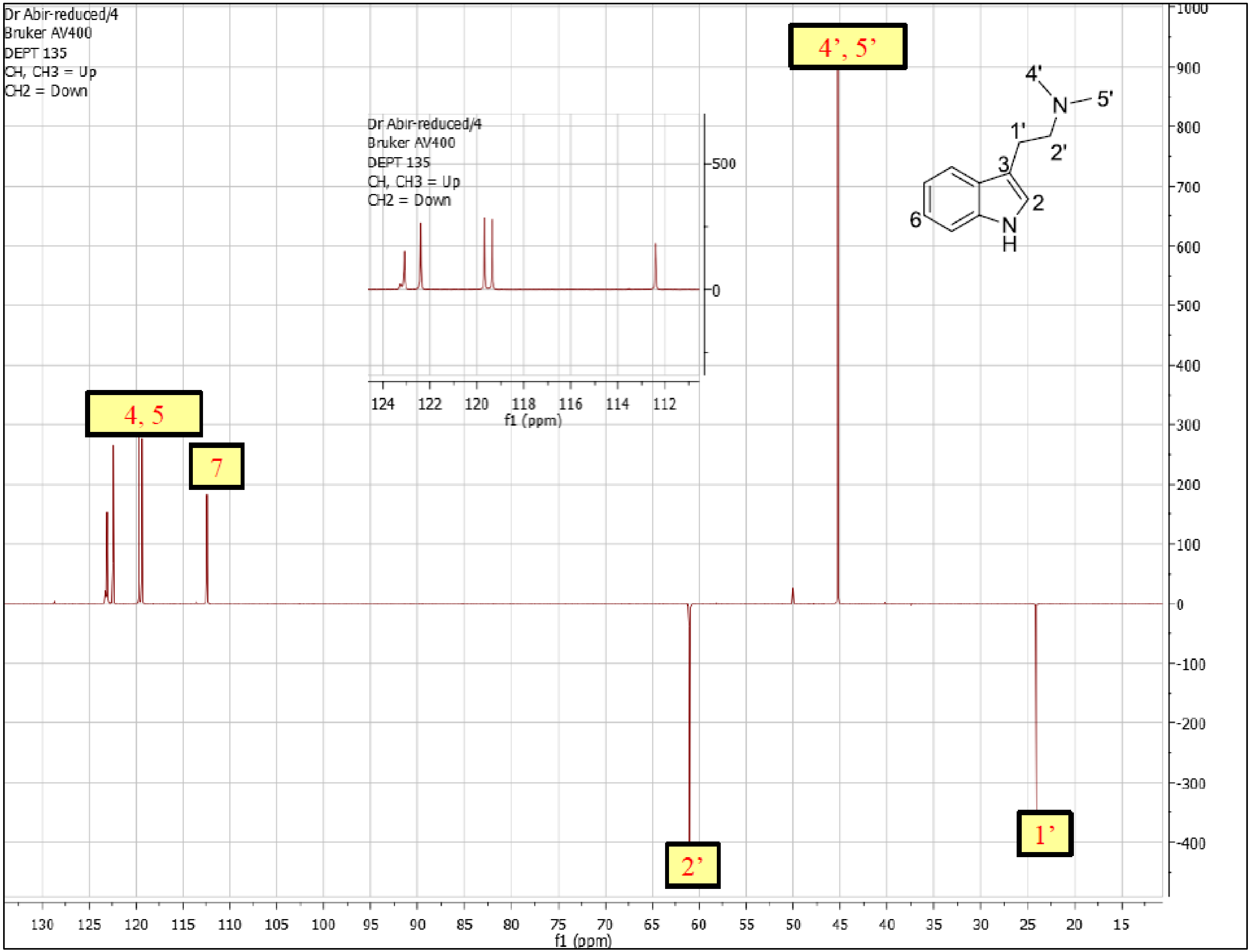

Figure 13. 135º DEPT spectrum of 2-(1*H*-indol-3-yl)-*N*,*N*-dimethylethanamine (**2a**) in methanol-d_4 (400 MHz)

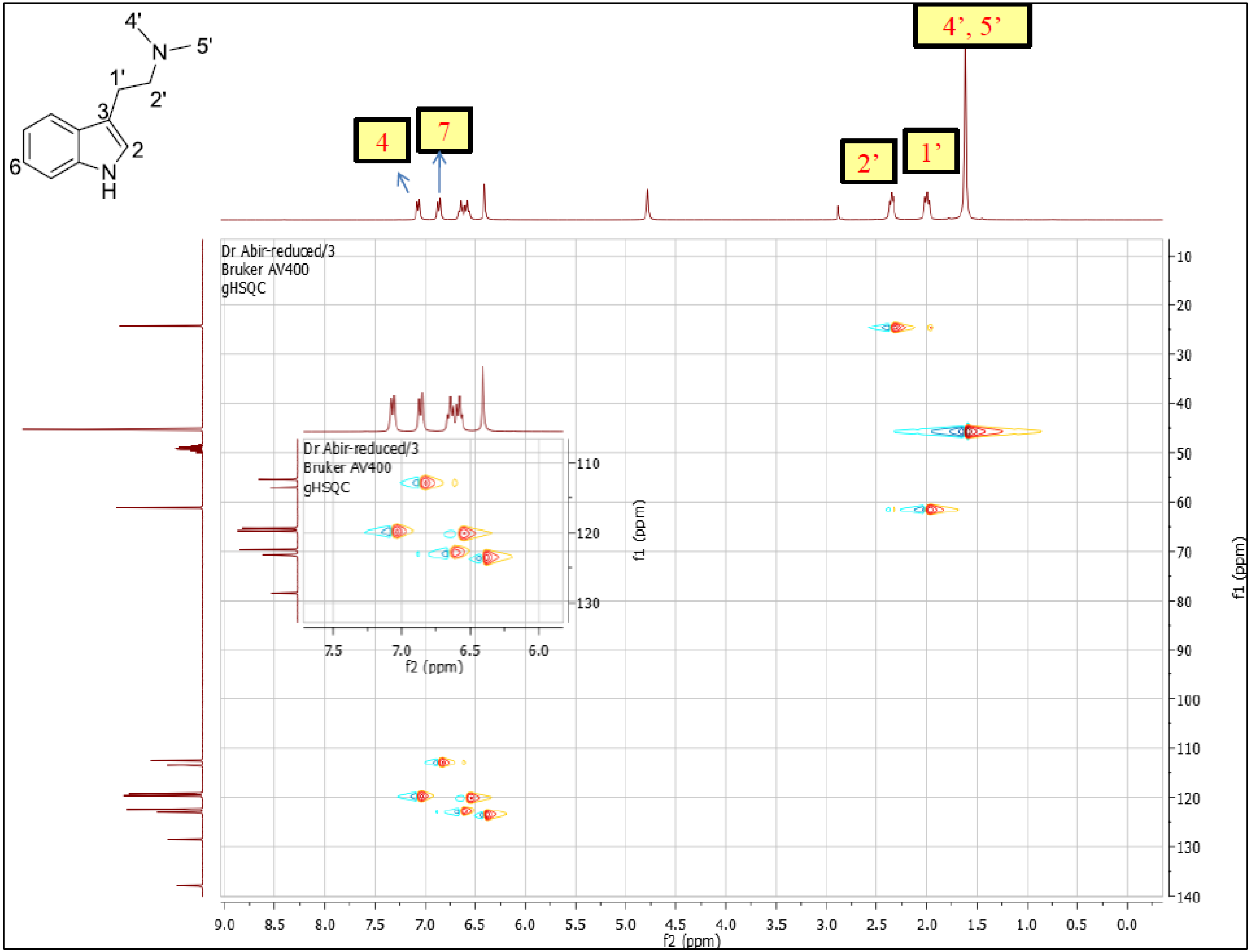

Figure 14. HSQC spectrum of 2-(1*H*-indol-3-yl)-*N*,*N*-dimethylethanamine (**2a**) in methanol-d_4 (400 MHz)

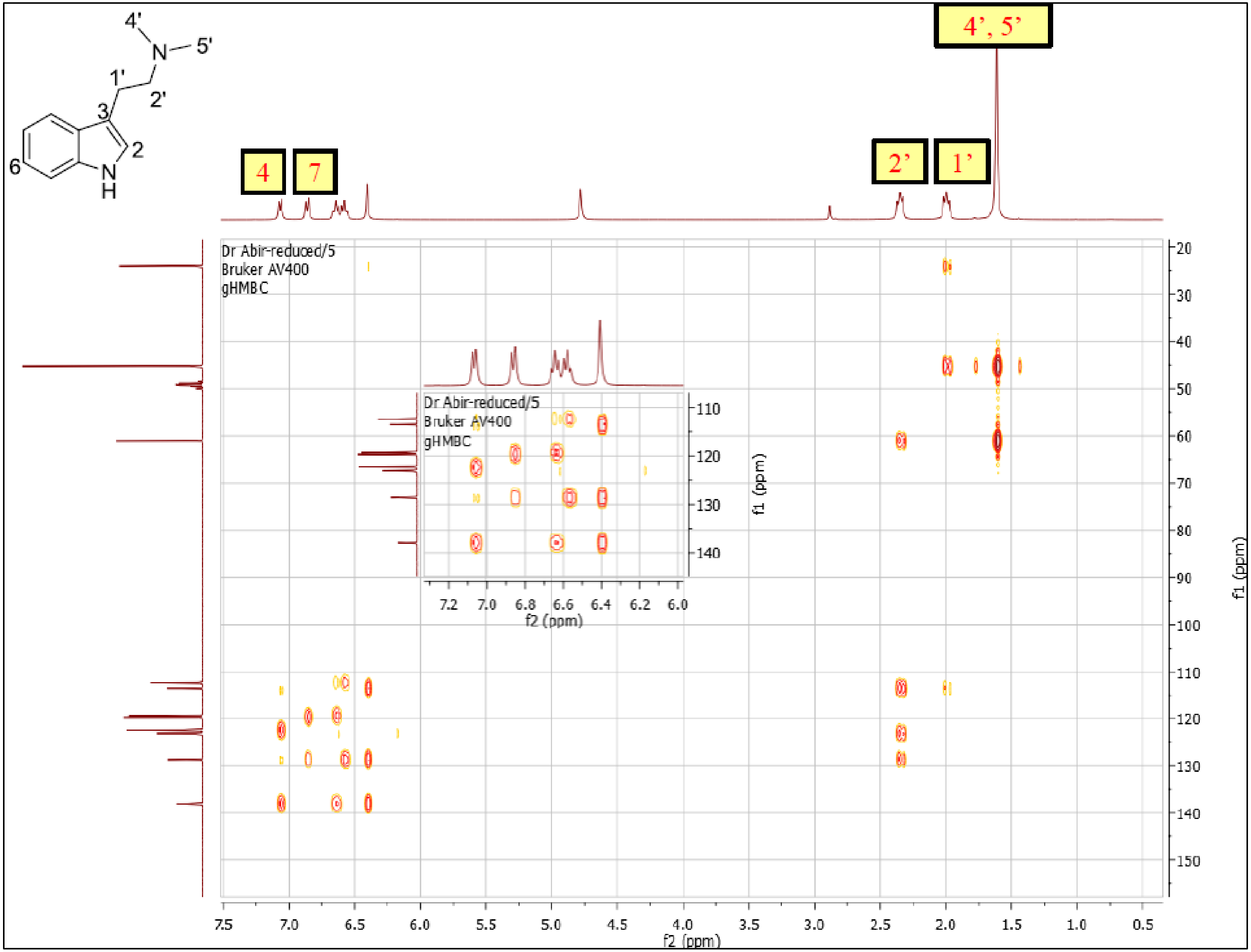

Figure 15. HMBC spectrum of 2-(1*H*-indol-3-yl)-*N*,*N*-dimethylethanamine (**2a**) in methanol-d_4 (400 MHz)

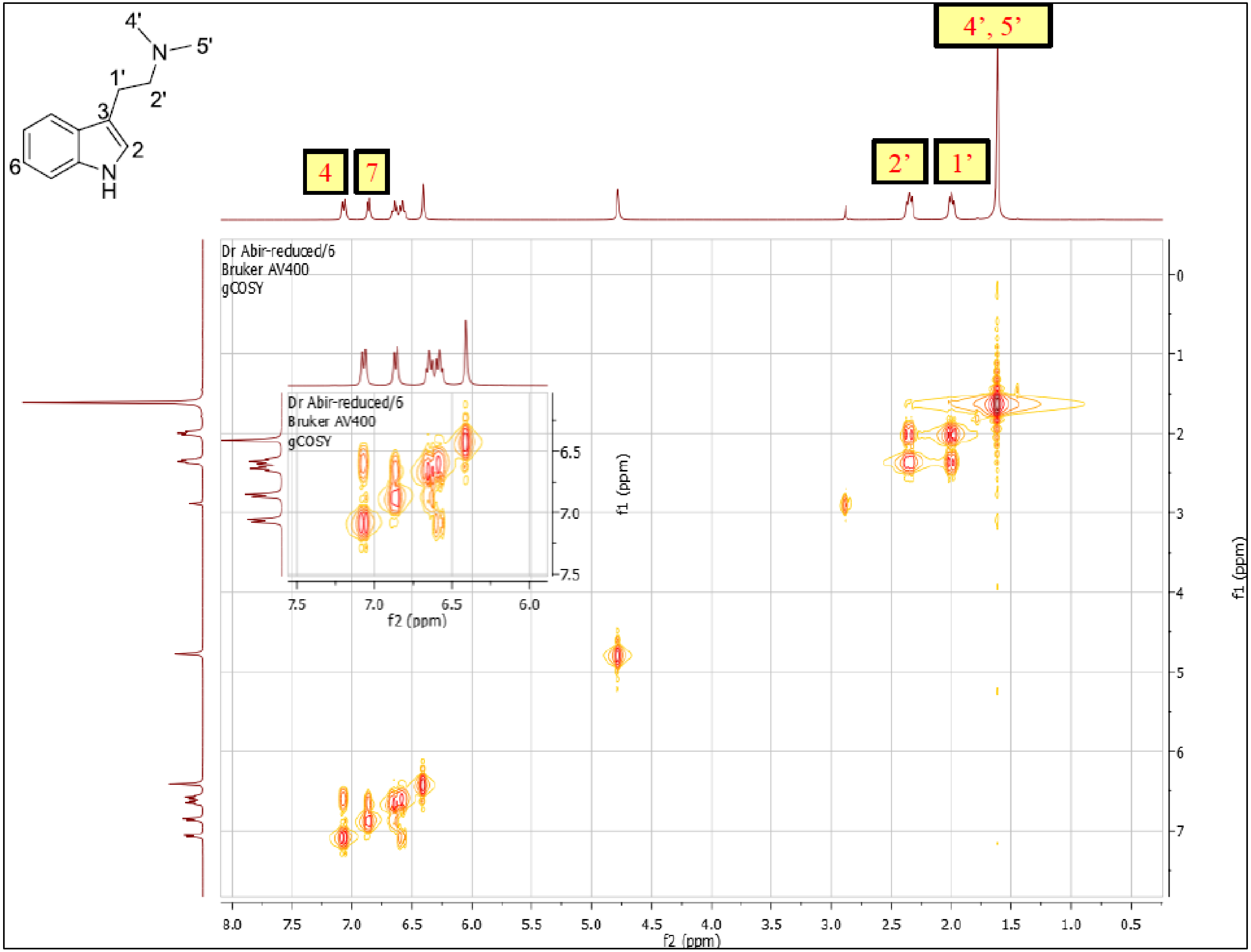

Figure 16. COSY spectrum of 2-(1*H*-indol-3-yl)-*N*,*N*-dimethylethanamine (**2a**) in methanol-d_4 (400 MHz)

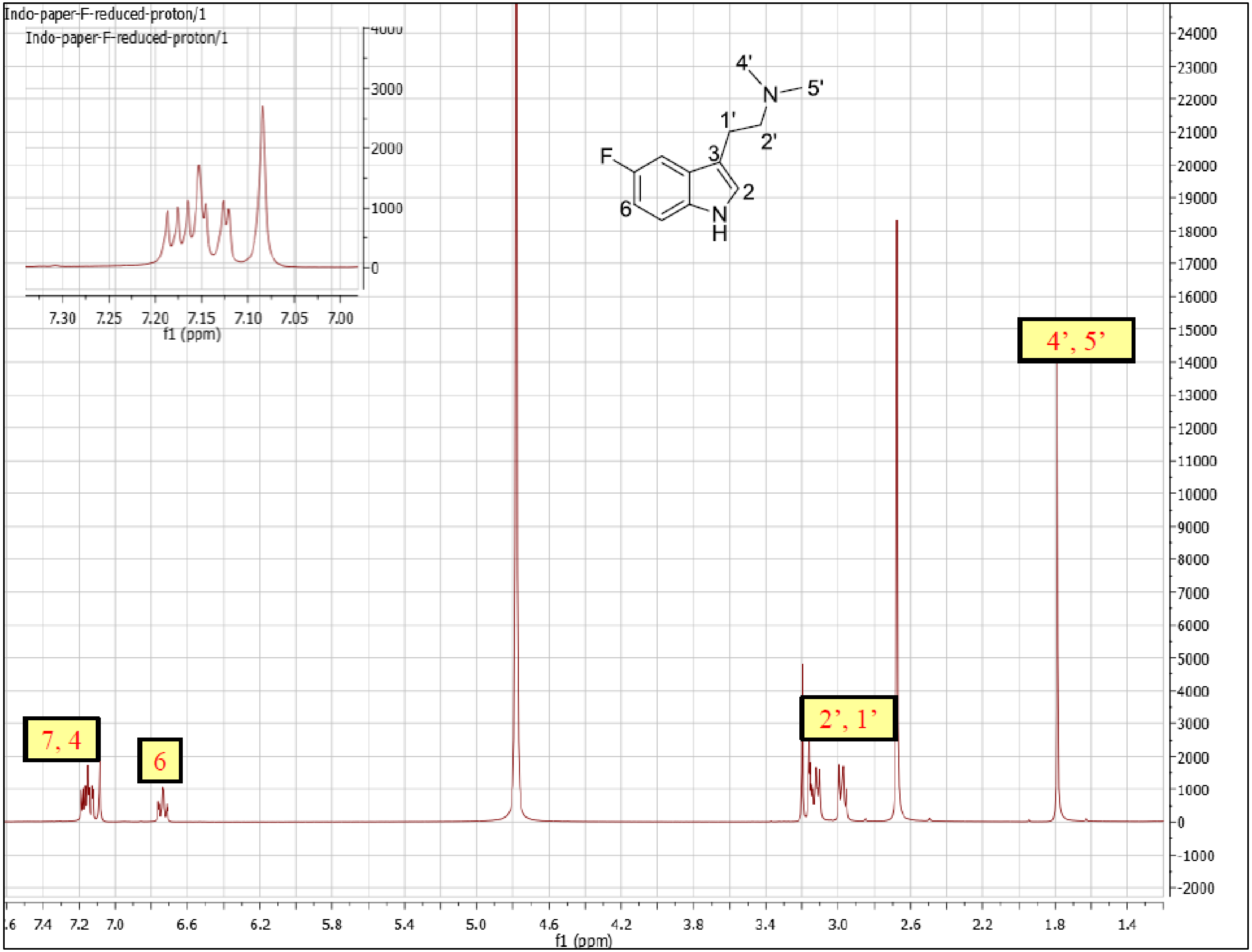

Figure 17. ¹H NMR spectrum of 2-(5-fluoro-1*H*-indol-3-yl)-*N*,*N*-dimethylethanamine (**2b**) in methanol-d_4 (400 MHz)

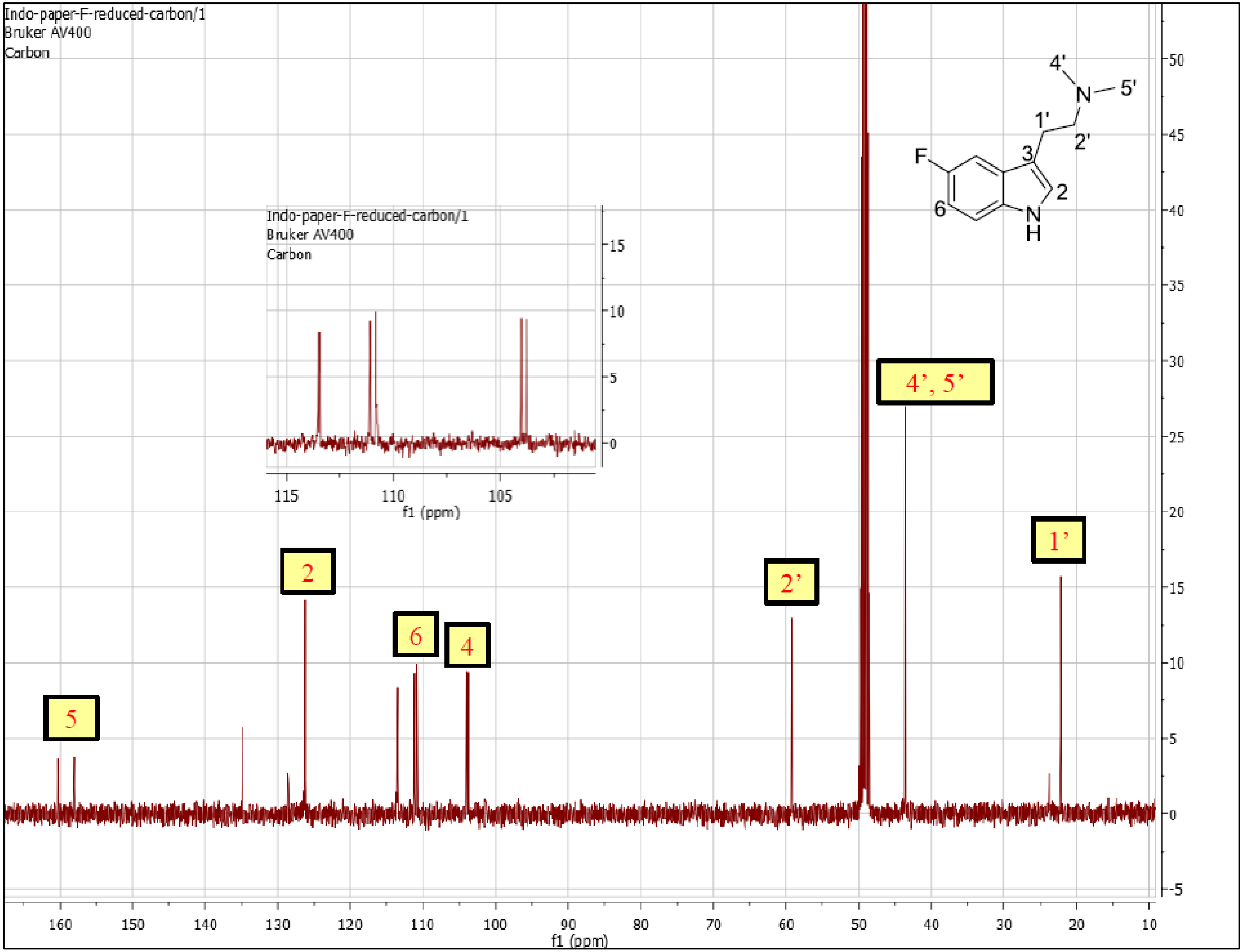

Figure 18. ^{13}C NMR spectrum of 2-(5-fluoro-1*H*-indol-3-yl)-*N*,*N*-dimethylethanamine (**2b**) in methanol-d_4 (400 MHz)

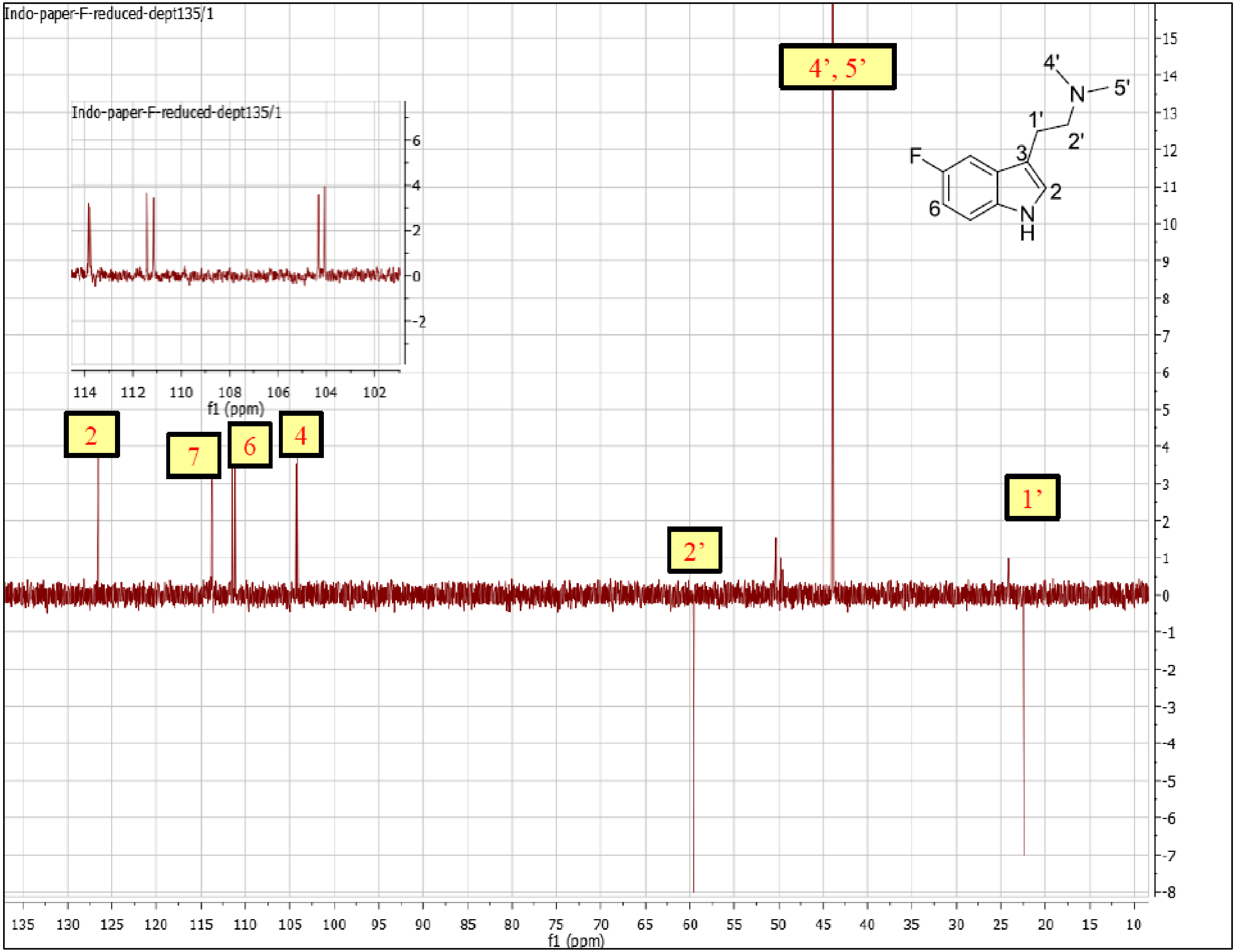

Figure 19. 135º DEPT spectrum of 2-(5-fluoro-1*H*-indol-3-yl)-*N*,*N*-dimethylethanamine (**2b**) in methanol-d_4 (400 MHz)

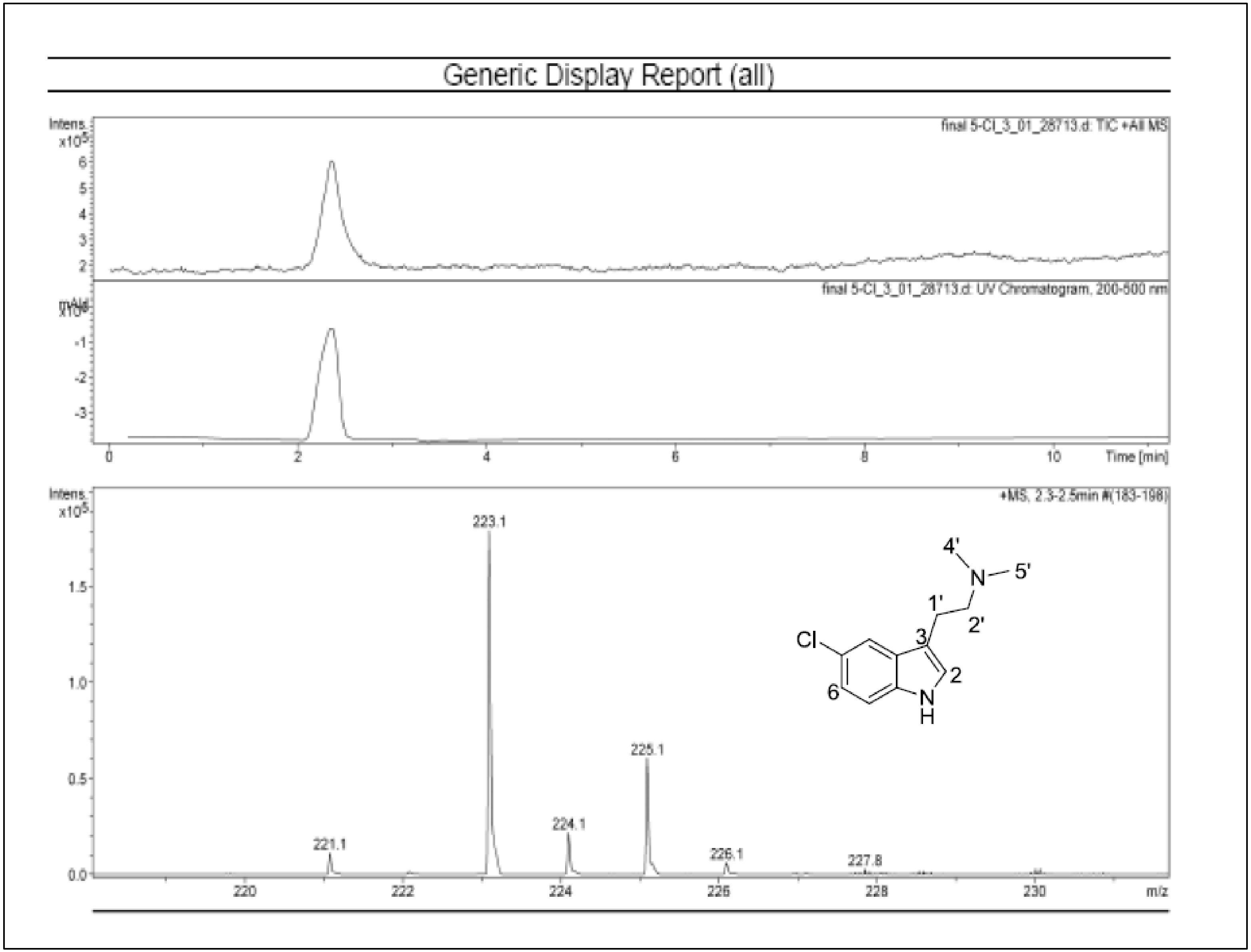

Figure 20. LC/MS chromatogram of 2-(5-chloro-1*H*-indol-3-yl)-*N*,*N*-dimethylethanamine (**2c**)

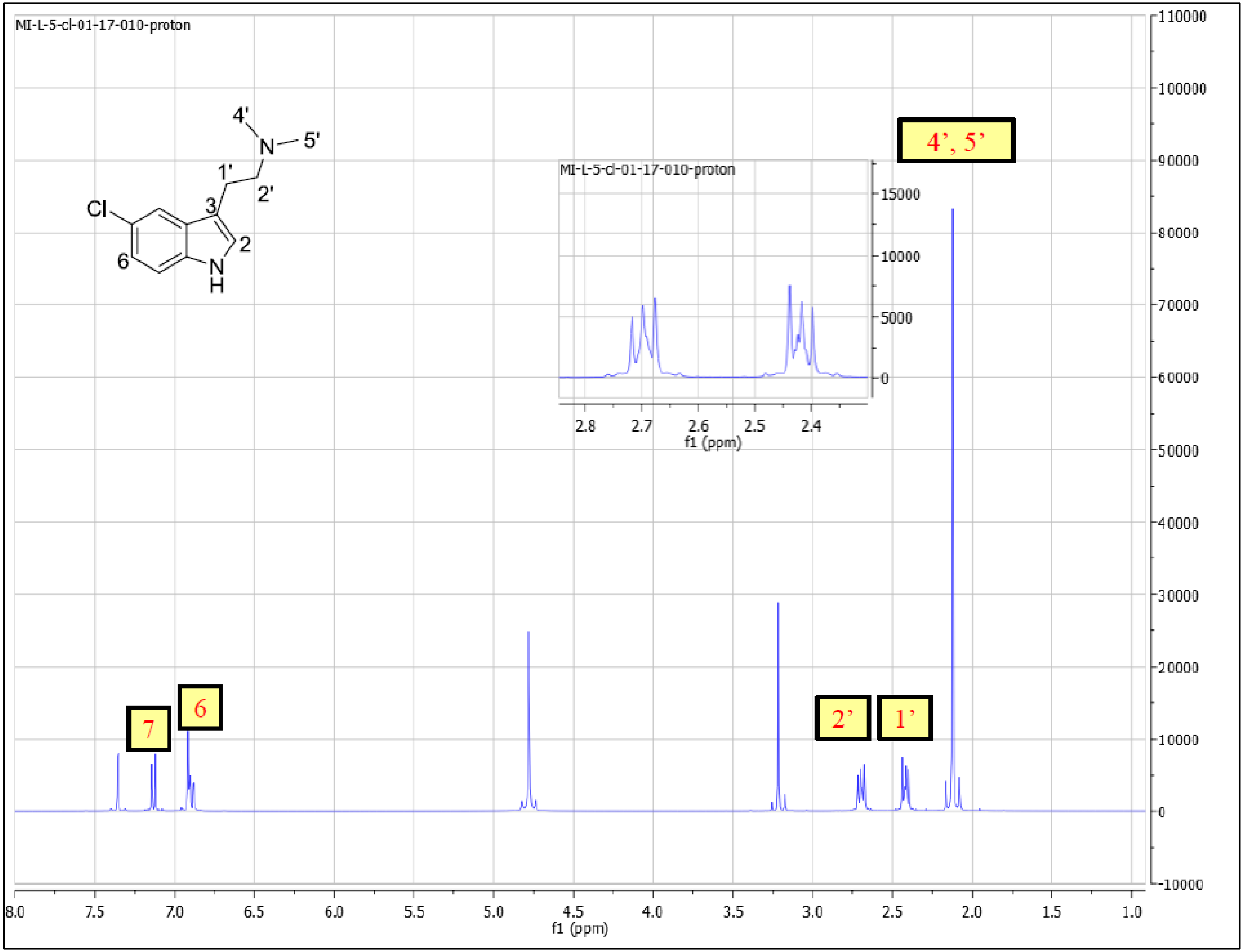

Figure 21. ^{1}H NMR spectrum of 2-(5-chloro-1*H*-indol-3-yl)-*N*,*N*-dimethylethanamine (**2c**) in methanol-d_4 (400 MHz)

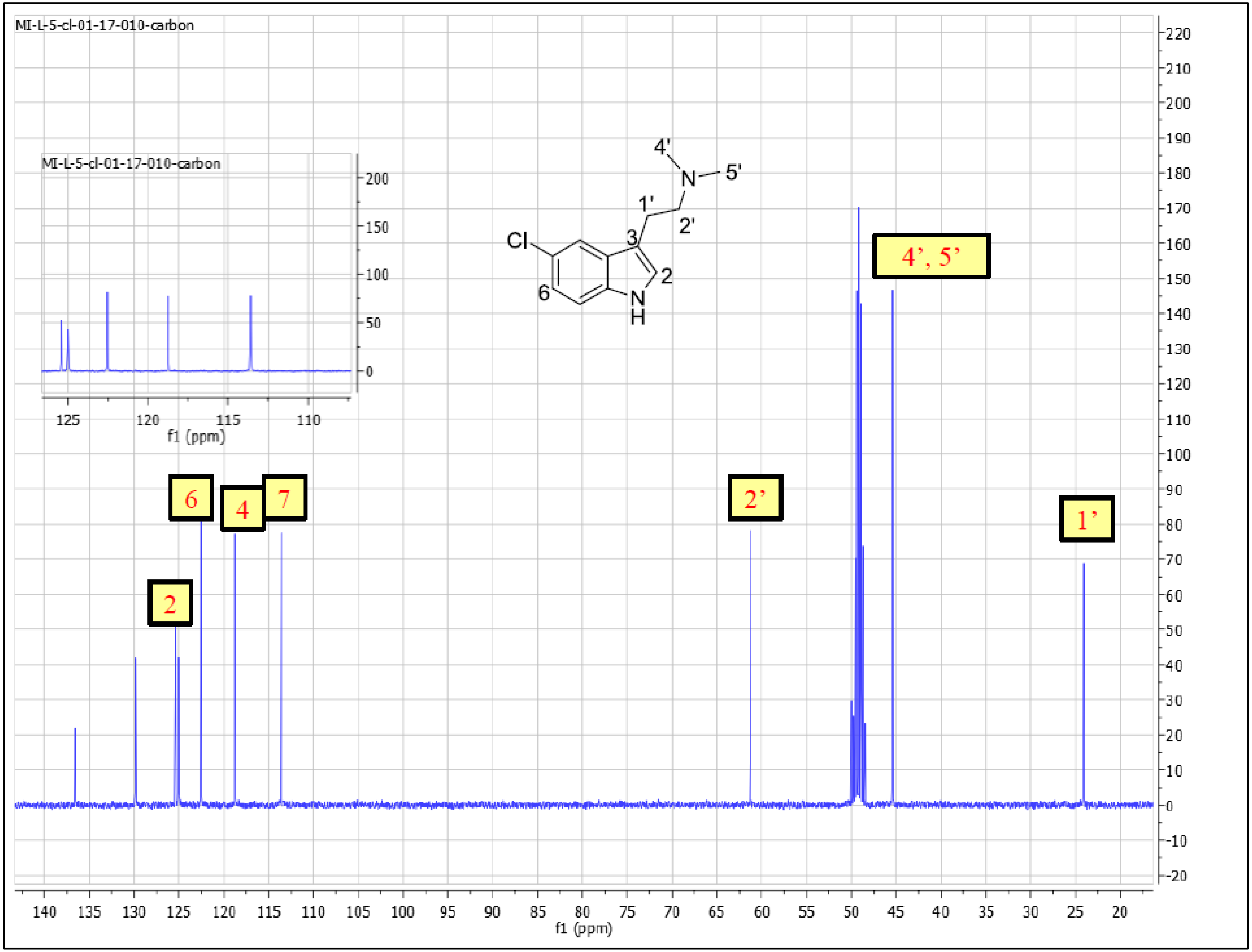

Figure 22. ^{13}C NMR spectrum of 2-(5-chloro-1*H*-indol-3-yl)-*N*,*N*-dimethylethanamine (**2c**) in methanol-d_4 (400 MHz)

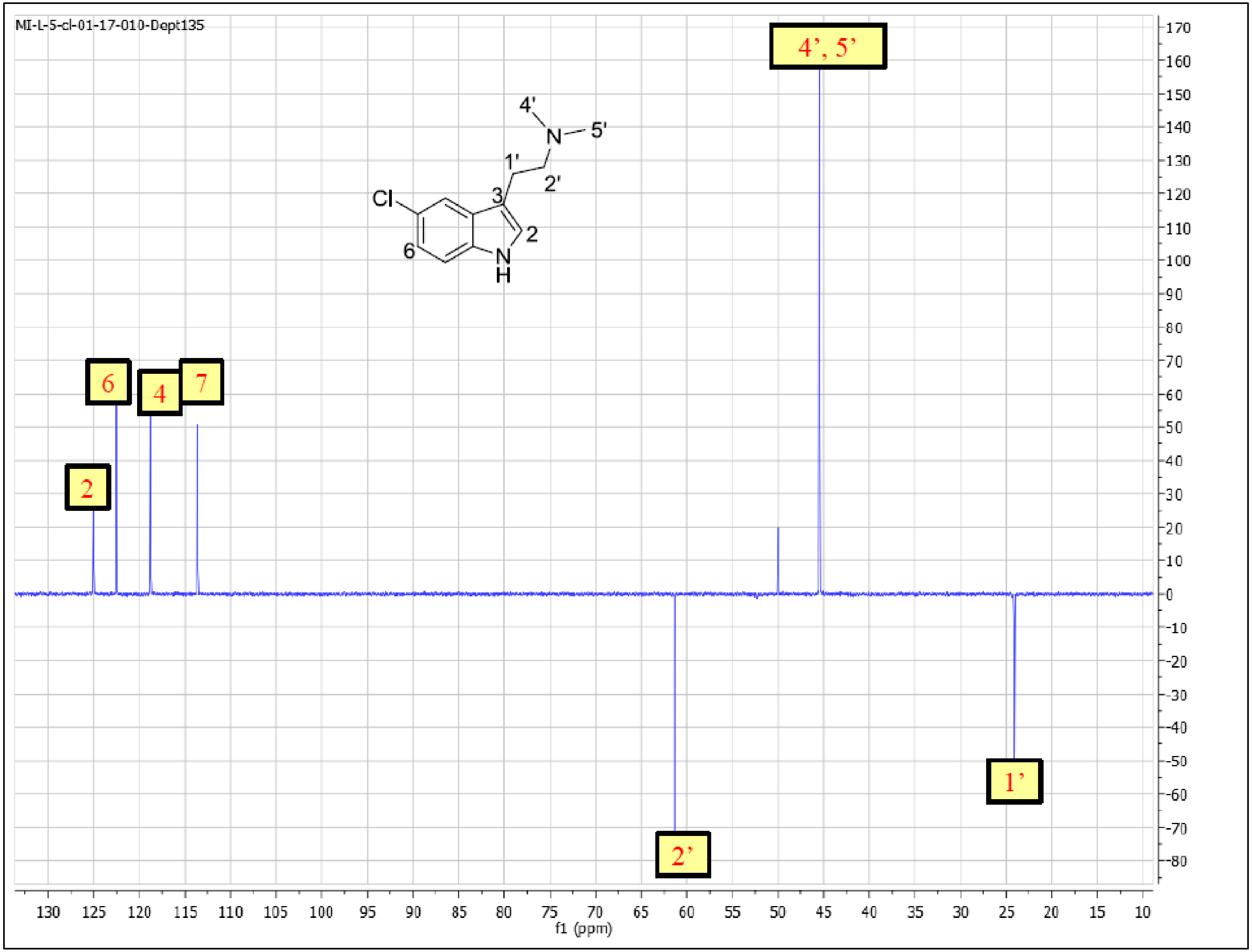

Figure 23. 135º DEPT spectrum of 2-(5-chloro-1*H*-indol-3-yl)-*N*,*N*-dimethylethanamine (**2c**) in methanol-d_4 (400 MHz)

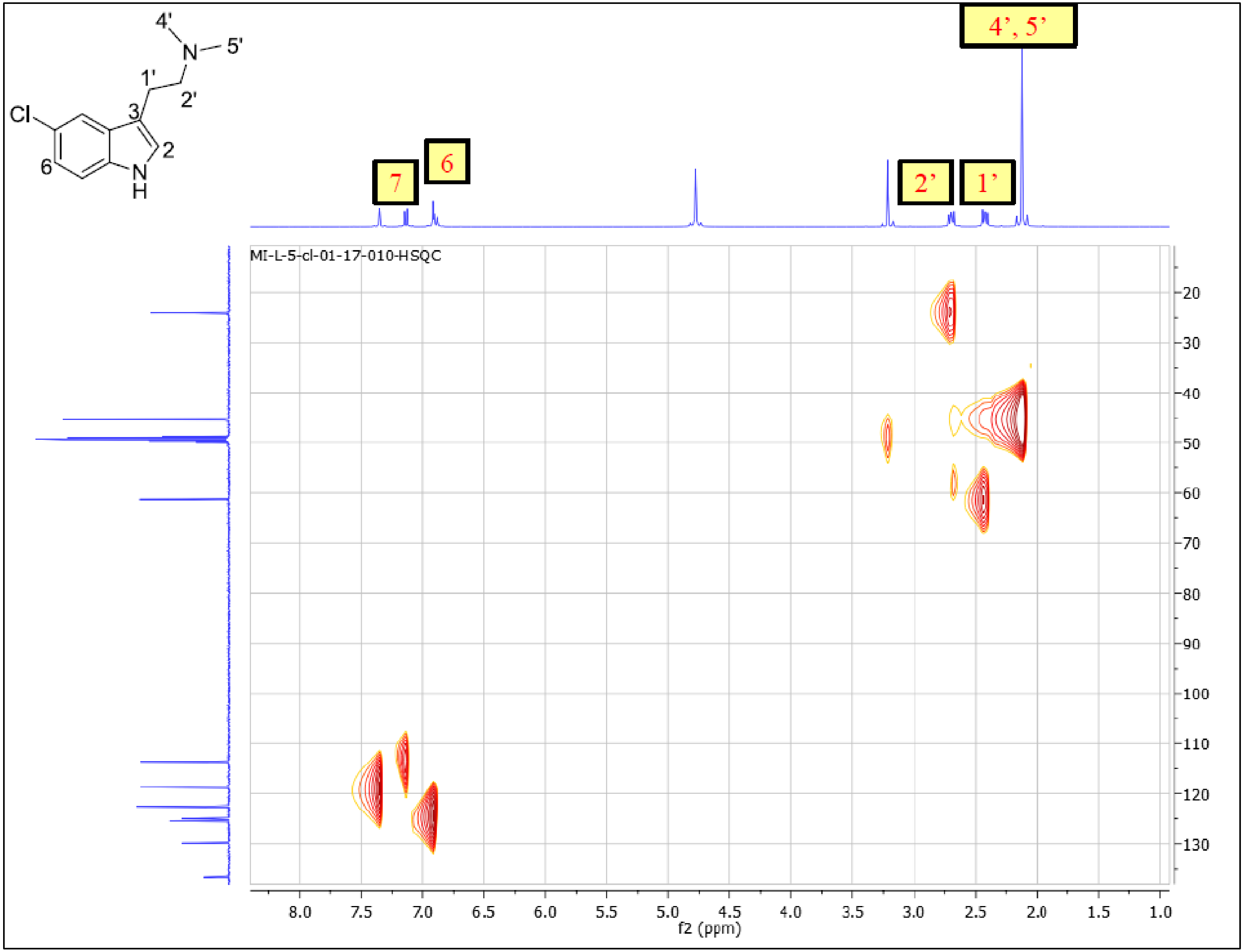

Figure 24. HSQC spectrum of 2-(5-chloro-1*H*-indol-3-yl)-*N*,*N*-dimethylethanamine (**2c**) in methanol-d_4 (400 MHz)

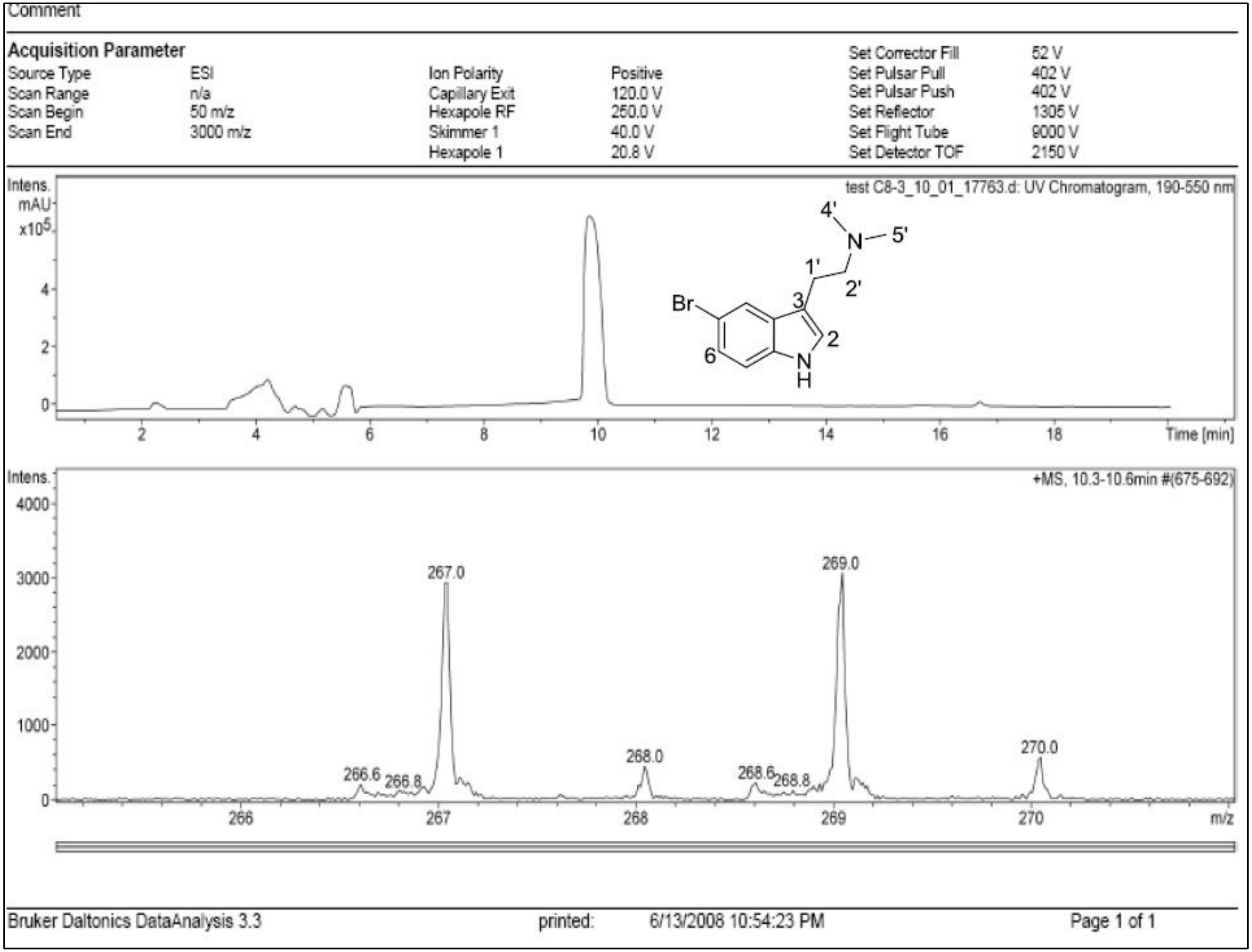

Figure 25. LC/MS chromatogram of 2-(5-bromo-1*H*-indol-3-yl)-*N*,*N*-dimethylethanamine (**2d**)

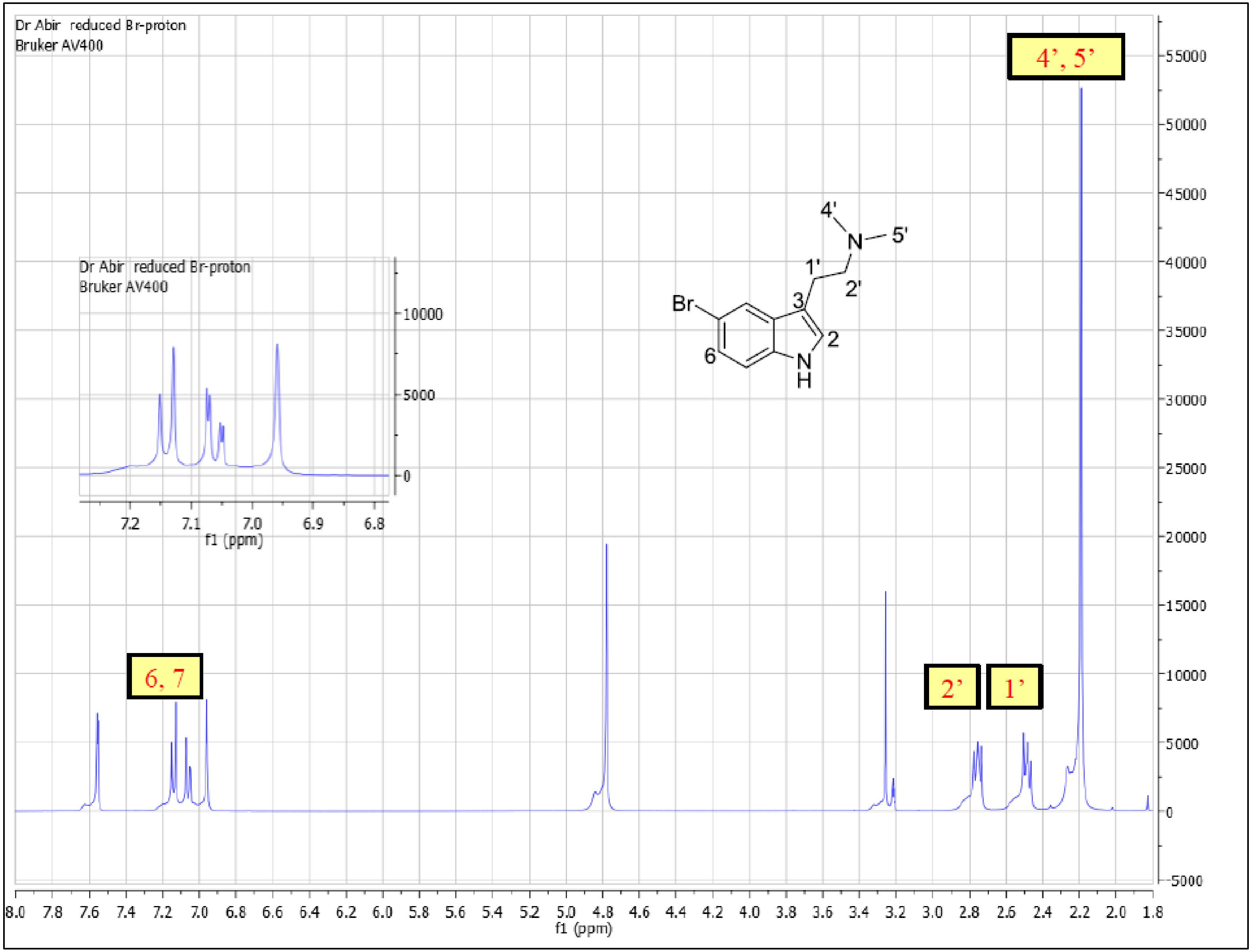

Figure 26. ^{1}H NMR spectrum of 2-(5-bromo-1*H*-indol-3-yl)-*N*,*N*-dimethylethanamine (**2d**) in methanol-d_4 (400 MHz)

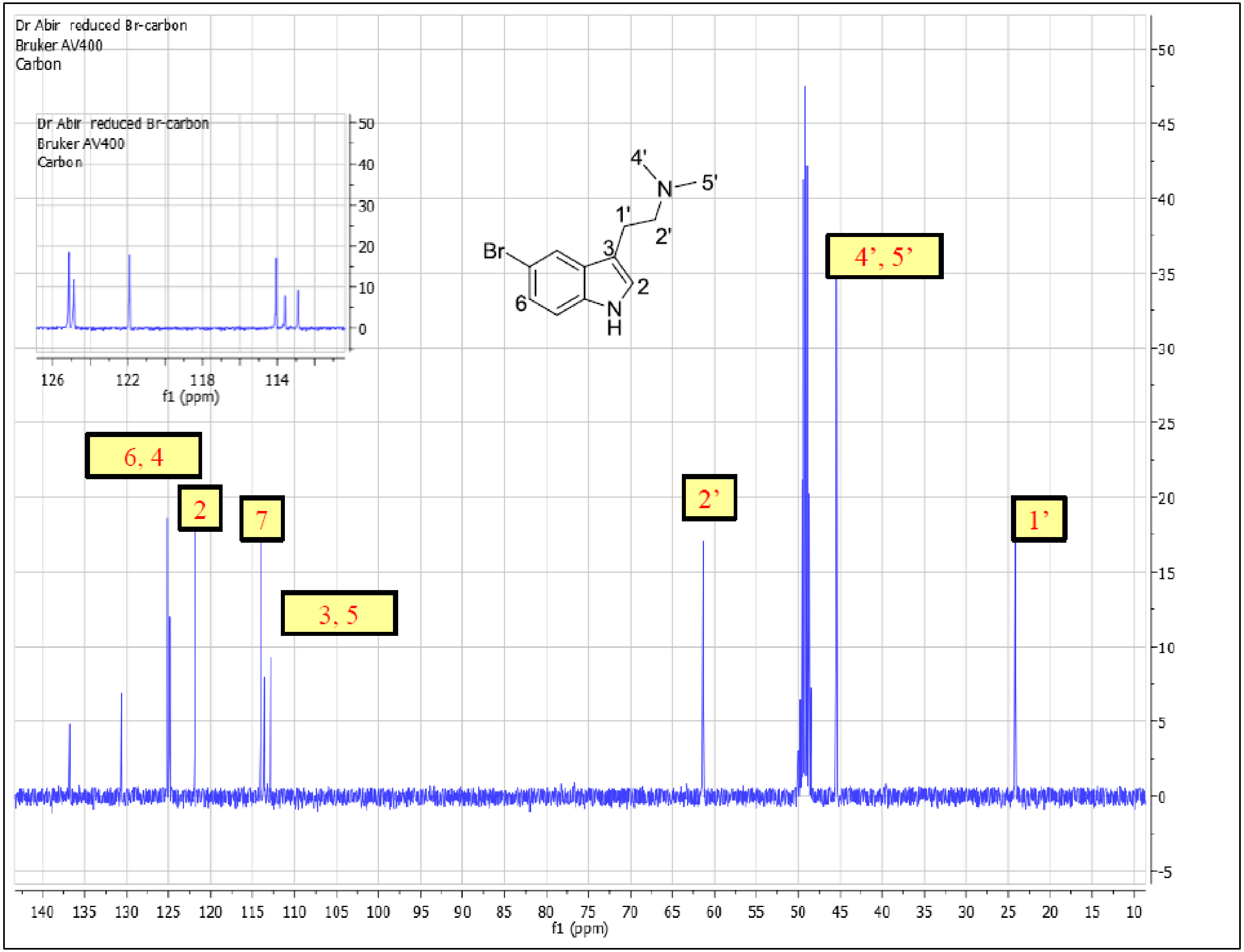

Figure 27. ^{13}C NMR spectrum of 2-(5-bromo-1*H*-indol-3-yl)-*N*,*N*-dimethylethanamine (**2d**) in methanol-d_4 (400 MHz)

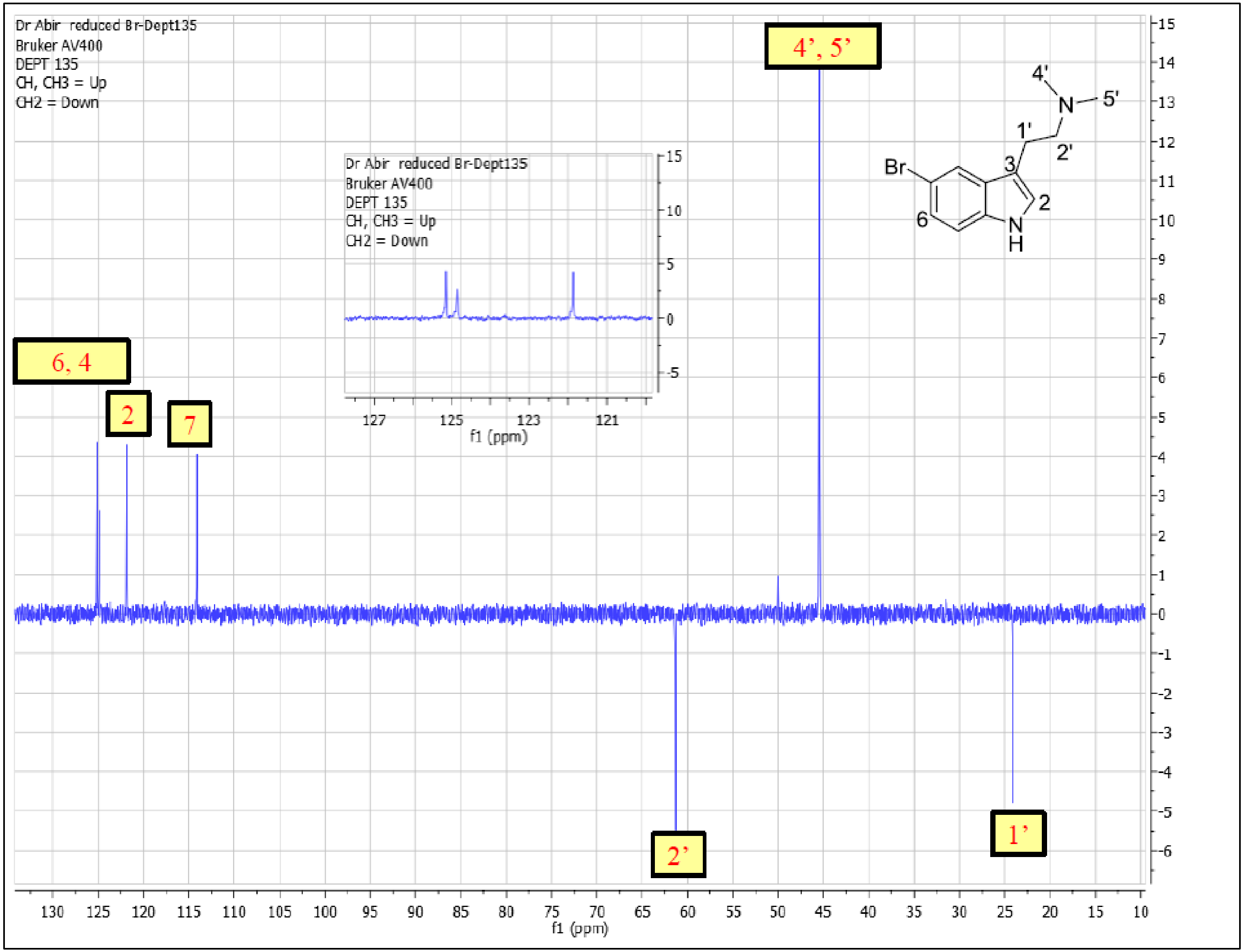

Figure 28. 135º DEPT spectrum of 2-(5-bromo-1*H*-indol-3-yl)-*N*,*N*-dimethylethanamine (**2d**) in methanol-d_4 (400 MHz)

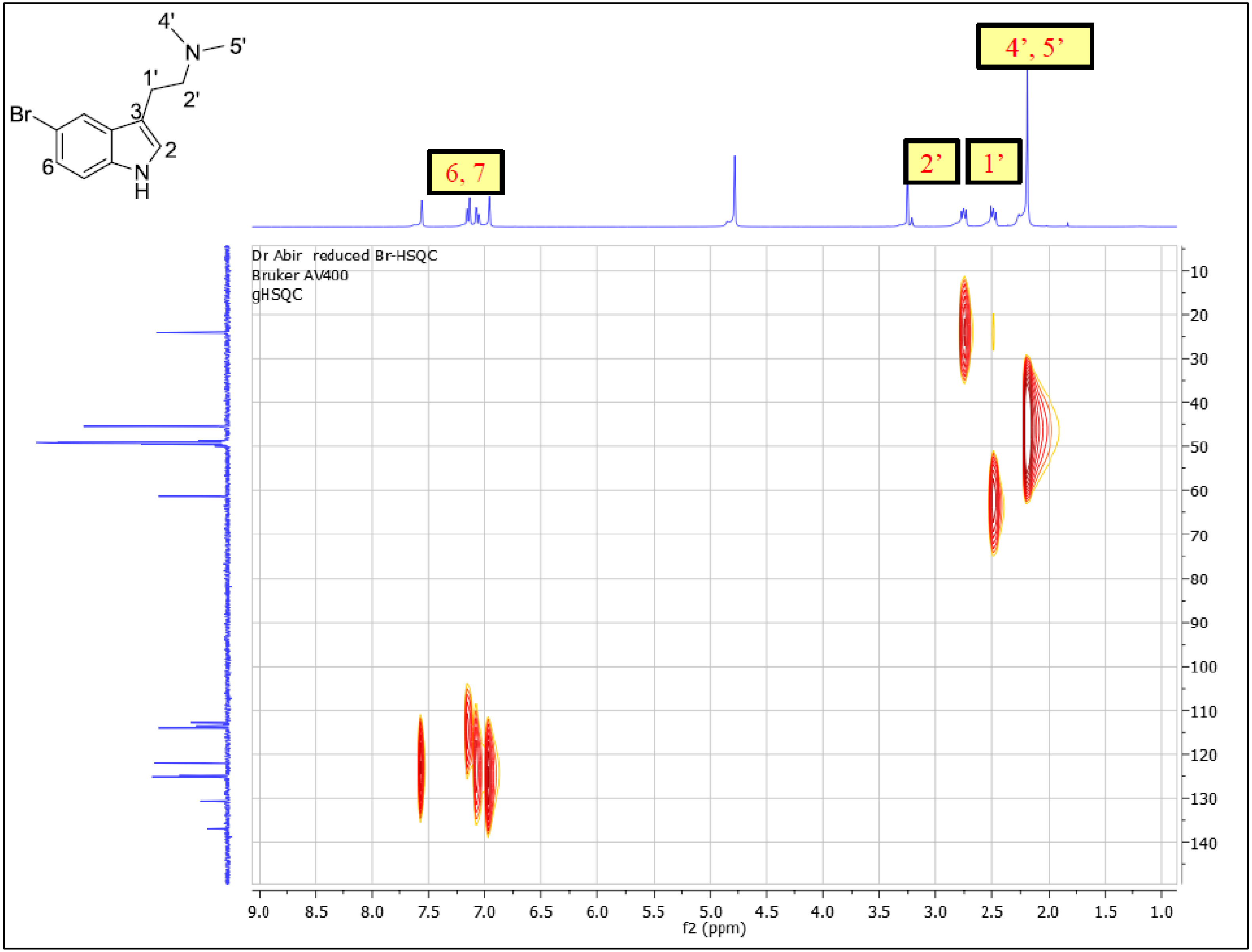

Figure 29. HSQC spectrum of 2-(5-bromo-1*H*-indol-3-yl)-*N*,*N*-dimethylethanamine (**2d**) in methanol-d_4 (400 MHz)

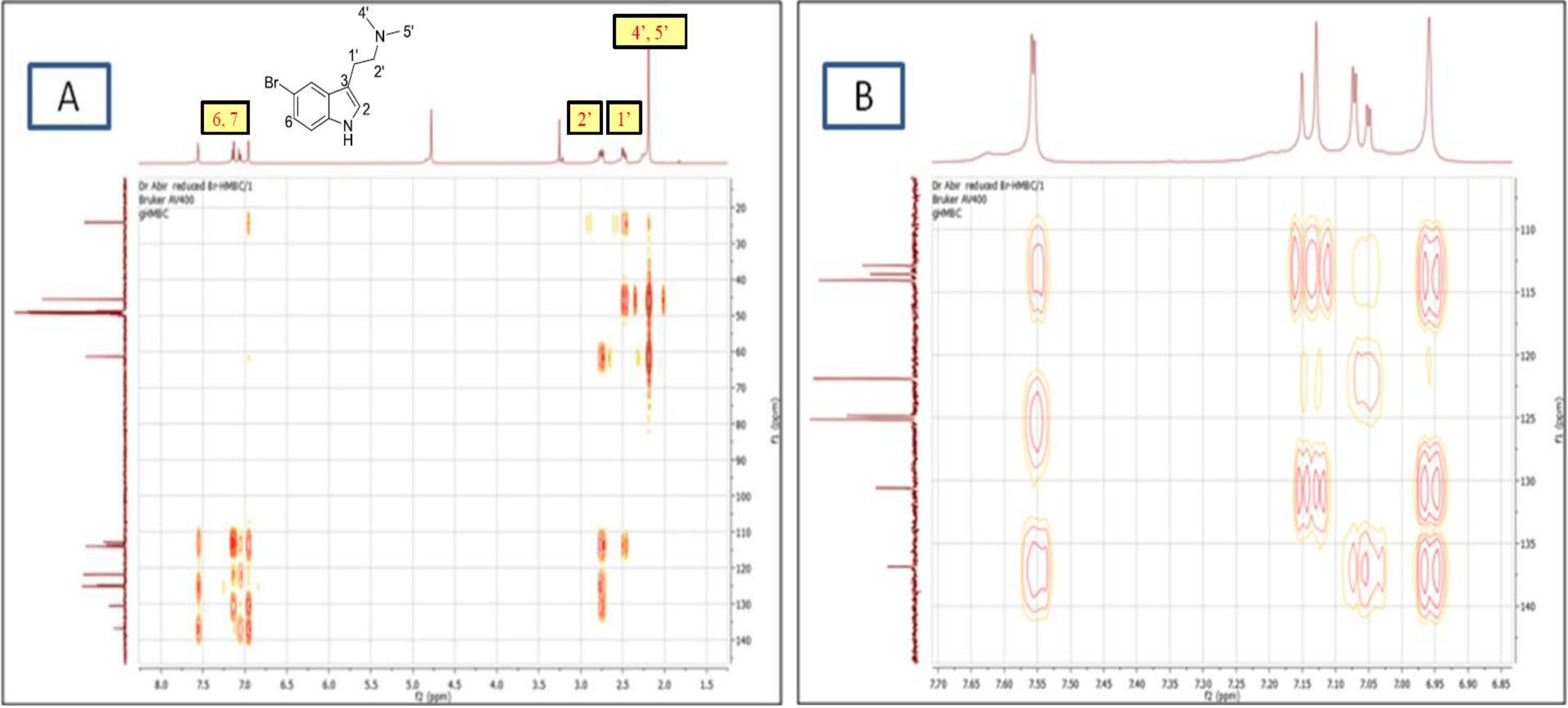

Figure 30. HMBC spectrum of 2-(5-bromo-1*H*-indol-3-yl)-*N*,*N*-dimethylethanamine (**2d**) in methanol-d_4 (400 MHz); A) the full spectrum, B) the high field region

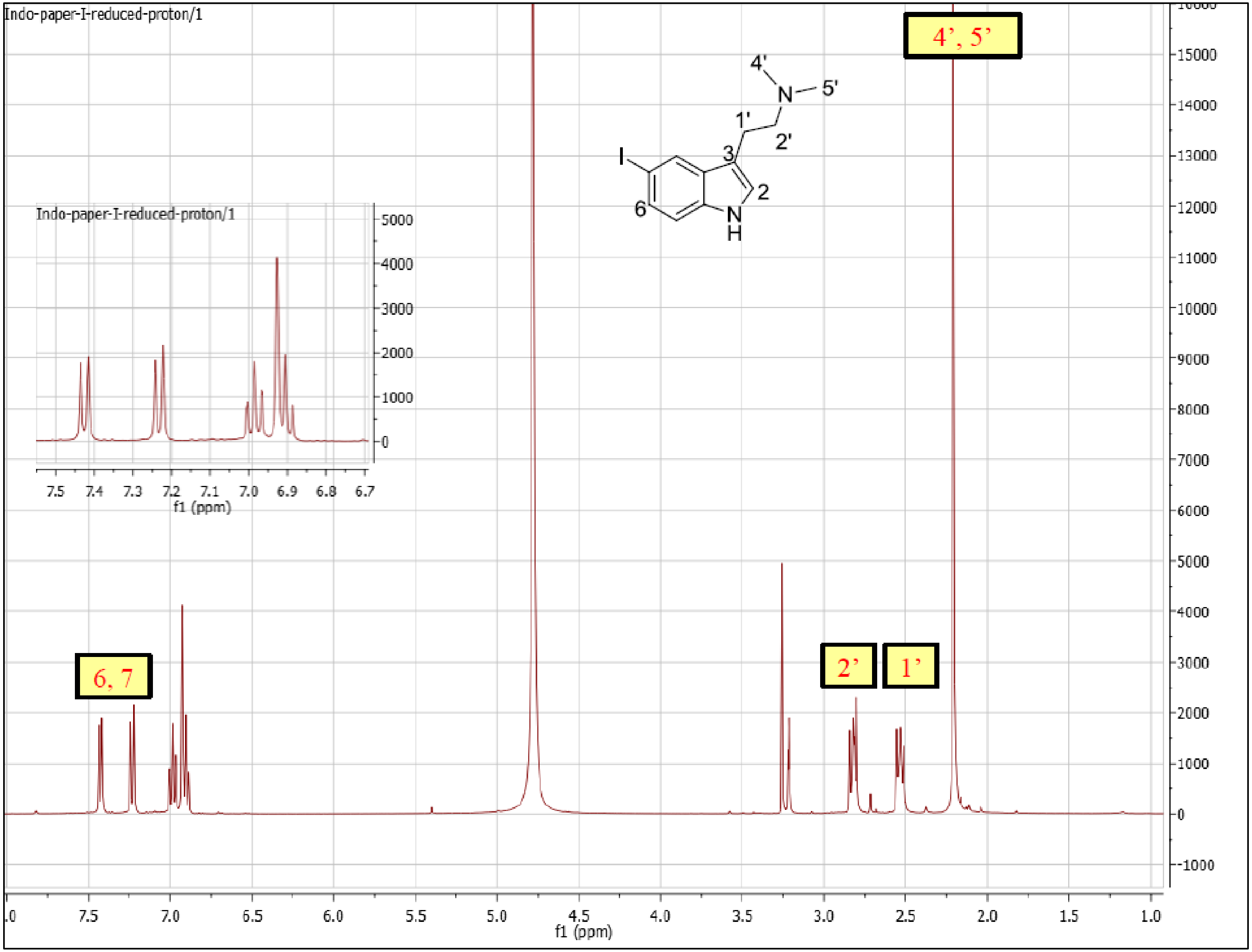

Figure 31. ^{1}H NMR spectrum of 2-(5-iodo-1*H*-indol-3-yl)-*N*,*N*-dimethylethanamine (**2e**) in methanol-d_4 (400 MHz)

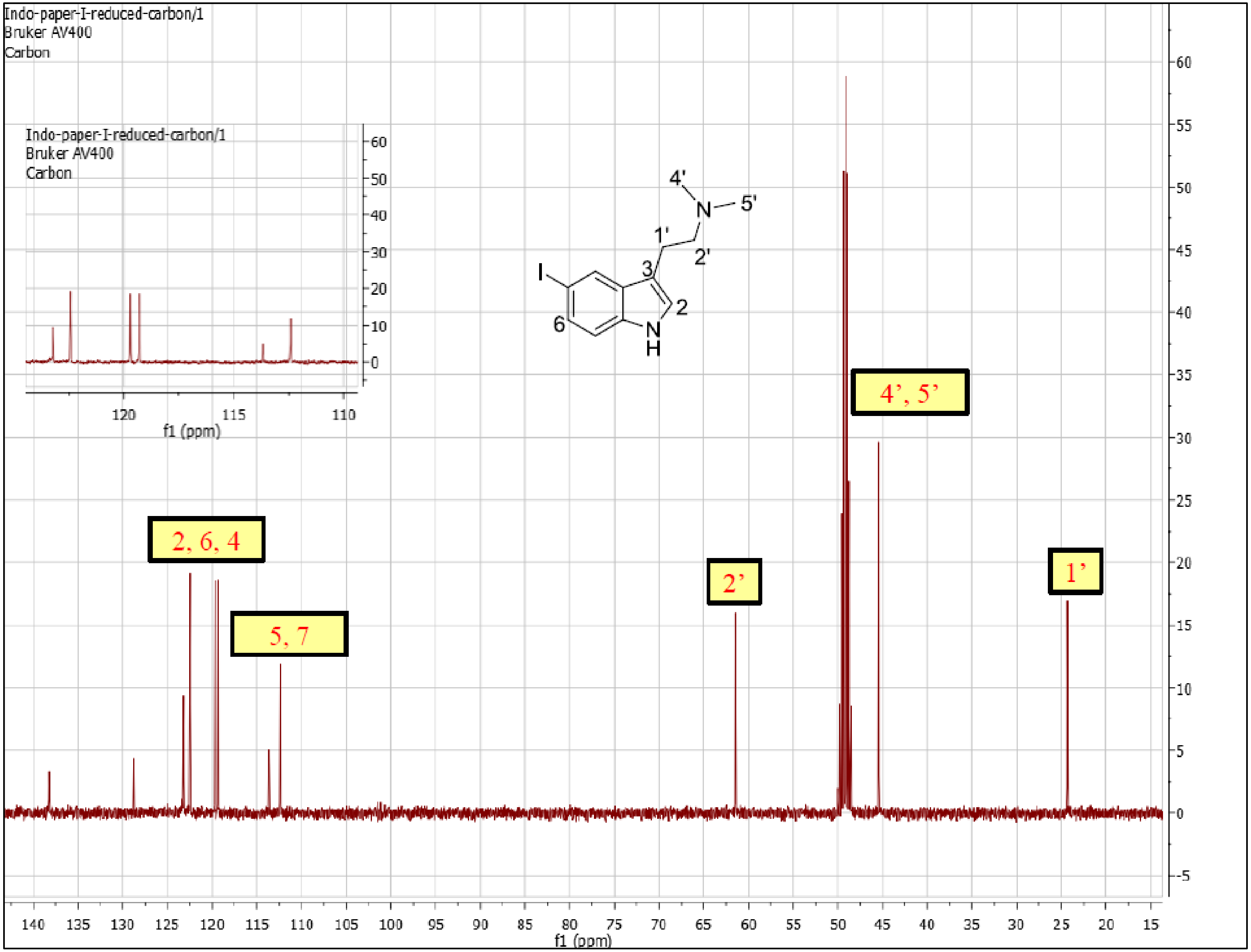

Figure 32. ^{13}C NMR spectrum of 2-(5-iodo-1*H*-indol-3-yl)-*N*,*N*-dimethylethanamine (**2e**) in methanol-d_4 (400 MHz)

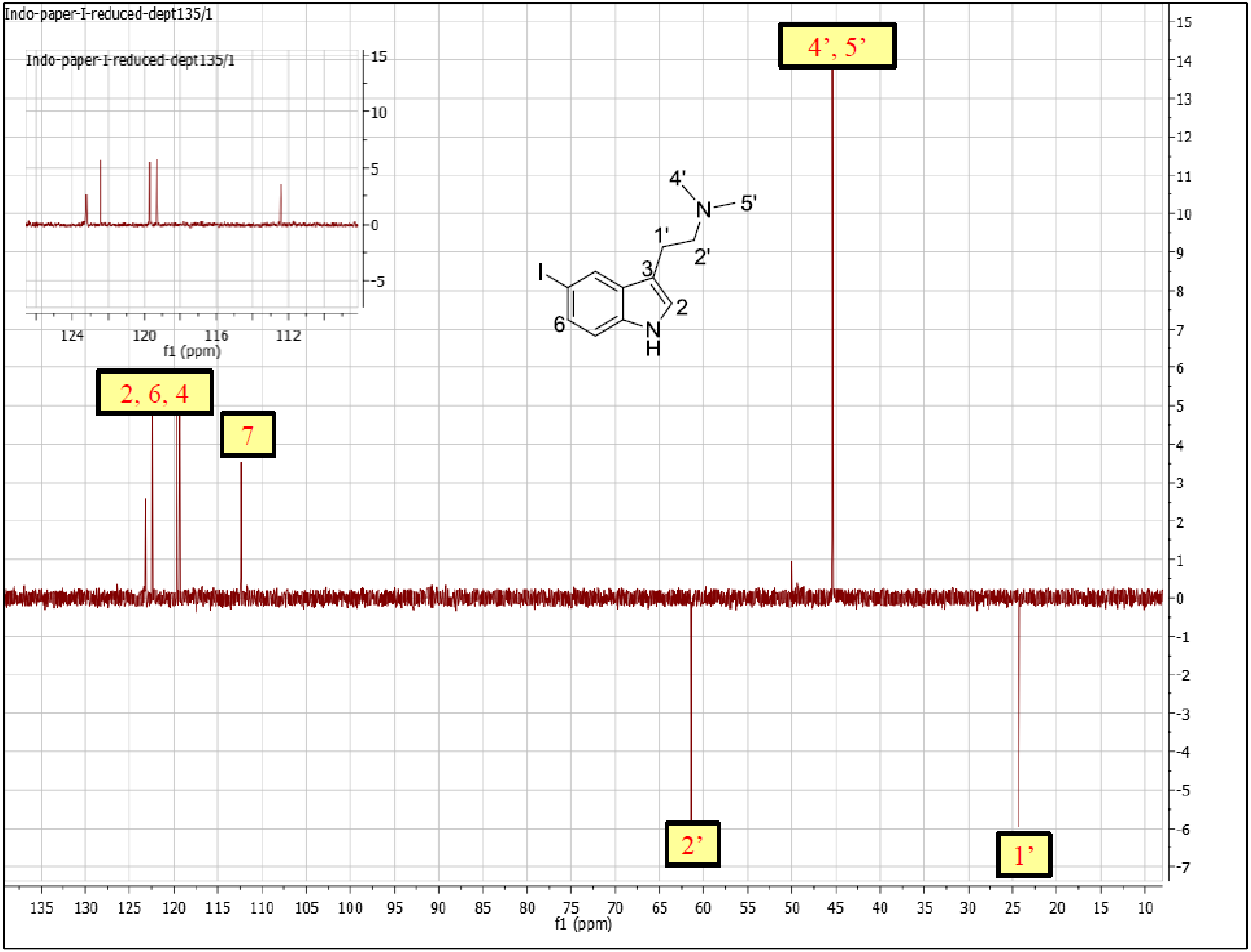

Figure 33. 135º DEPT spectrum of 2-(5-iodo-1*H*-indol-3-yl)-*N*,*N*-dimethylethanamine (**2e**) in methanol-d_4 (400 MHz)
